# Hyper-parallel nonlocal CNOT operation with hyperentanglement assisted by cross-Kerr nonlinearity

**DOI:** 10.1038/s41598-019-52173-x

**Published:** 2019-11-04

**Authors:** Ping Zhou, Li Lv

**Affiliations:** 1 College of Science, Guangxi University for Nationalities, Nanning, 530006 People’s Republic of China; 2 Key lab of quantum information and quantum optics, Guangxi University for Nationalities, Nanning, 530006 People’s Republic of China; 3 Guangxi Key Laboratory of Hybrid Computational and IC Design Analysis, Nanning, 530006 People’s Republic of China

**Keywords:** Quantum optics, Quantum information, Quantum optics

## Abstract

Implementing CNOT operation nonlocally is one of central tasks in distributed quantum computation. Most of previously protocols for implementation quantum CNOT operation only consider implement CNOT operation in one degree of freedom(DOF). In this paper, we present a scheme for nonlocal implementation of hyper-parallel CNOT operation in polarization and spatial-mode DOFs via hyperentanglement. The CNOT operations in polarization DOF and spatial-mode DOF can be remote implemented simultaneously with hyperentanglement assisited by cross-Kerr nonlinearity. Hyper-parallel nonlocal CNOT gate can enhance the quantum channel capacity for distributed quantum computation and long-distance quantum communication. We discuss the experiment feasibility for hyper-parallel nonlocal gate. It shows that the protocol for hyper-parallel nonlocal CNOT operation can be realized with current technology.

## Introduction

The application of quantum entangled state and quantum superposition state in quantum information processing allows the agents in quantum communication to exploit the quantum mechanical phenomena to transmit quantum information securely^1–14^, provides quantum computing with computation power which has no counterpart in classical computing^15–22^. Researchers devoted much interest in realization of quantum computation via different physical systems, such as cavity quantum electrodynamics (QED)^23,24^, quantum dots^25–28^, linear-optical systems^29–31^, nuclear magnetic resonance(NMR)^32,33^, trapped ions^34–38^ and superconducting qubits^39,40^. The realization of large-scale quantum computation requires storage and manipulations of the large number of qubits. Implementing distributed quantum computation via nodes in quantum network which is connected by entangled quantum channel provides a method to scale up the number of quantum qubits in quantum computation since the number of qubits stored and manipulated in a single quantum system could be limited in practical application^41^.

Implementing quantum operation on remote quantum system is one of central tasks in distributed quantum computation. It is shown that universal quantum gate can be constructed by single-qubit gates and two-qubit controlled-not(CNOT) gates, or constructed by single-qubit gates and three-qubit Toffoli gates^42^. Remote implementation of quantum operations has attached much interest^43–54^. On the one hand, theoretical schemes for remote implementation of quantum operations, especially single-qubit operations, two-qubit CNOT operations and three-qubit gates, have been presented via different quantum channels. In 1999, Gottesman and Chuang studied university quantum computation via quantum gate teleportation^43^. The CNOT operation can be teleported from acting on local qubits to acting on remote qubits by using entangled state $|\chi \rangle =\frac{1}{2}[(|00\rangle +|11\rangle )|00\rangle +(|01\rangle +|10\rangle )|11\rangle ]$ as the quantum channel. In 2000, Eisert *et al*. discussed the minimal resources required in remote implementation of nonlocal quantum operations^44^. They showed that quantum CNOT operation can be nonlocal implemented via two bits of classical communication and one ebit of quantum entanglement (a maximally entangled state of two qubits). Jiang *et al*. presented a scheme for deterministic remote implementation of nonlocal coupling gates between different registers^45^. In 2013, Wang *et al*. proposed a scheme for teleportation of quantum CNOT gate via quantum dots^46^. In 2015, Hu *et al*. proposed a protocol for deterministic remote implementation of nonlocal Toffoli operation among distant solid-state qubits^47^. In 2018, Lv *et al*. presented a scheme for multiparty joint remote implementation of an arbitrary single-qubit operation via single-qubit measurements and quantum entangled channel^48^. On the other hand, remote implementation of nonlocal CNOT operation between two physical qubits^52^ or two logical qubits^53^, nonlocal implementation of quantum controlled-SWAP gate have been experimental demonstrated^54^.

Quantum states simultaneously entangled in multiple degrees of freedom(DOF) is quantum hyperentangled state^55^. In the past few years, quantum information processing via hyperentanglement has attached much interest since its high channel capacity. Hyperentangled state is used to assist complete Bell-state analysis^56^, hyperentanglement Bell-state analysis^57^ and deterministic entanglement purification^58^ in one DOF. Quantum hyperteleportation^59^ and hyperentanglement swapping^60^ can be realized with hyperentangled Bell state analysis. Quantum states in different DOFs can be parallel remote prepared by using hyperentangled state as the quantum channel^61^. Quantum hyperentangled state can also be used to construct universal hyperparallel quantum gates for quantum computation^62,63^. Quantum superdense coding via hyperentanglement can beat the channel capacity for long-distance communication^64^. Moreover, protocols for hyperentanglement purification^65^ and concentration^66^ are proposed for high-capacity long-distance quantum communication via hyperentangled states.

The previously protocols for nonlocal remote implementation of two-qubit CNOT operation only consider implement quantum operation in one DOF^47,48^. Different from previously protocols, we present a protocol for parallel nonlocal implementation of the CNOT operation both in polarization DOF and in spatial-mode DOF by using a two-photon four-qubit hyperentangled state as the quantum channel. Assisted by cross-Kerr nonlinearity, hyperentangled state, the CNOT operation can be teleported from acting on local qubits in polarization and spatial-mode DOFs to acting on remote qubits in two DOFs. The agent first applies CNOT operation in polarization DOF via polarization entanglement of the hyperentangled state, then applies CNOT operation in spatial-mode DOF via spatial-mode entanglement of the hyperentangled state. We discussed the experimental feasibility for parallel nonlocal remote implementation of CNOT operations simultaneously and show that it is accessible with current technology.

## Results

### Nonlocal CNOT operation in the polarization DOF

To present the principle of our protocol for hyper-parallel CNOT gate clearly, we first present the protocol for nonlocal implementation of CNOT operation in polarization DOF via cross-Kerr nonlinearity, then present the protocol for nonlocal implementation of CNOT operation in spatial-mode DOF.

Similar to the case for nonlocal remote implementation of CNOT operation in one DOF, the agent Alice has the photon *A*_1_ whose polarization state and spatial-mode state are arbitrary single-qubit states

1 $$|\psi {\rangle }_{{A}_{1}}=({\alpha }_{1}|H\rangle +{\beta }_{1}|V\rangle )\otimes ({\gamma }_{1}|{a}_{1}\rangle +{\delta }_{1}|{b}_{1}\rangle ),$$

where $|H\rangle$, $|V\rangle$ represent horizontal polarization and vertical polarization of photon *A*_1_, $|{a}_{1}\rangle$, $|{b}_{1}\rangle$ are two spatial modes of photon *A*_1_. The complex coefficients *α*_1_, *β*_1_, *γ*_1_, *δ*_1_ satisfy the normalization conditions: $|{\alpha }_{1}{|}^{2}+|{\beta }_{1}{|}^{2}=1$, $|{\gamma }_{1}{|}^{2}+|{\delta }_{1}{|}^{2}=1$.

The agent Bob has photon *B*_1_ whose polarization state and spatial-mode state are arbitrary single-qubit states

2 $$|\psi {\rangle }_{{B}_{1}}=({\alpha }_{2}|H\rangle +{\beta }_{2}|V\rangle )\otimes ({\gamma }_{2}|{a}_{4}\rangle +{\delta }_{2}|{b}_{4}\rangle ).$$

Here $|{a}_{4}\rangle$, $|{b}_{4}\rangle$ are two spatial modes of photon *B*_1_. The complex coefficients *α*_2_, *β*_2_, *γ*_2_, *δ*_2_ satisfy the normalization conditions: $|{\alpha }_{2}{|}^{2}+|{\beta }_{2}{|}^{2}=1$, $|{\gamma }_{2}{|}^{2}+|{\delta }_{2}{|}^{2}=1$.

The two agents want to implement nonlocal CNOT operation on photons *A*_1_, *B*_1_ in polarization DOF by using polarization state of photon *A*_1_ as the control qubit, and implement nonlocal CNOT operation on photons *A*_1_, *B*_1_ in spatial-mode DOF by using the spatial-mode state of photon *A*_1_ as the control qubit.

To parallel implement nonlocal CNOT operations in polarization and spatial-mode DOFs, the two agents Alice and Bob share a two-photon four-qubit hyperentangled state $|\psi {\rangle }_{{A}_{2}{B}_{2}}$ as the quantum channel^67^. Here

3 $$|\psi {\rangle }_{{A}_{2}{B}_{2}}=\frac{1}{2}(|HH\rangle +|VV\rangle )\otimes (|{a}_{2}{a}_{3}\rangle +|{b}_{2}{b}_{3}\rangle ),$$

$|{a}_{2}\rangle$, $|{b}_{2}\rangle$ are two spatial modes of photon *A*_2_. $|{a}_{3}\rangle$, $|{b}_{3}\rangle$ represent two spatial modes of photon *B*_2_. Alice has photon *A*_2_ and Bob has photon *B*_2_,

The composite state of photons *A*_1_, *A*_2_, *B*_2_, *B*_1_ can be written as (neglect the whole factor $\tfrac{1}{2}$):

4 $$\begin{array}{rcl}|{\Psi }_{1}\rangle  & = & |\psi {\rangle }_{{A}_{1}}\otimes |\psi {\rangle }_{{A}_{2}{B}_{2}}\otimes |\psi {\rangle }_{{B}_{1}}\\  & = & ({\alpha }_{1}|H\rangle +{\beta }_{1}|V\rangle )(|HH\rangle +|VV\rangle )({\alpha }_{2}|H\rangle \\  &  & +\,{\beta }_{2}|V\rangle )({\gamma }_{1}|{a}_{1}\rangle +{\delta }_{1}|{b}_{1}\rangle )(|{a}_{2}{a}_{3}\rangle \\  &  & +\,|{b}_{2}{b}_{3}\rangle )({\gamma }_{2}|{a}_{4}\rangle +{\delta }_{2}|{b}_{4}\rangle ).\end{array}$$

**Figure 1:**
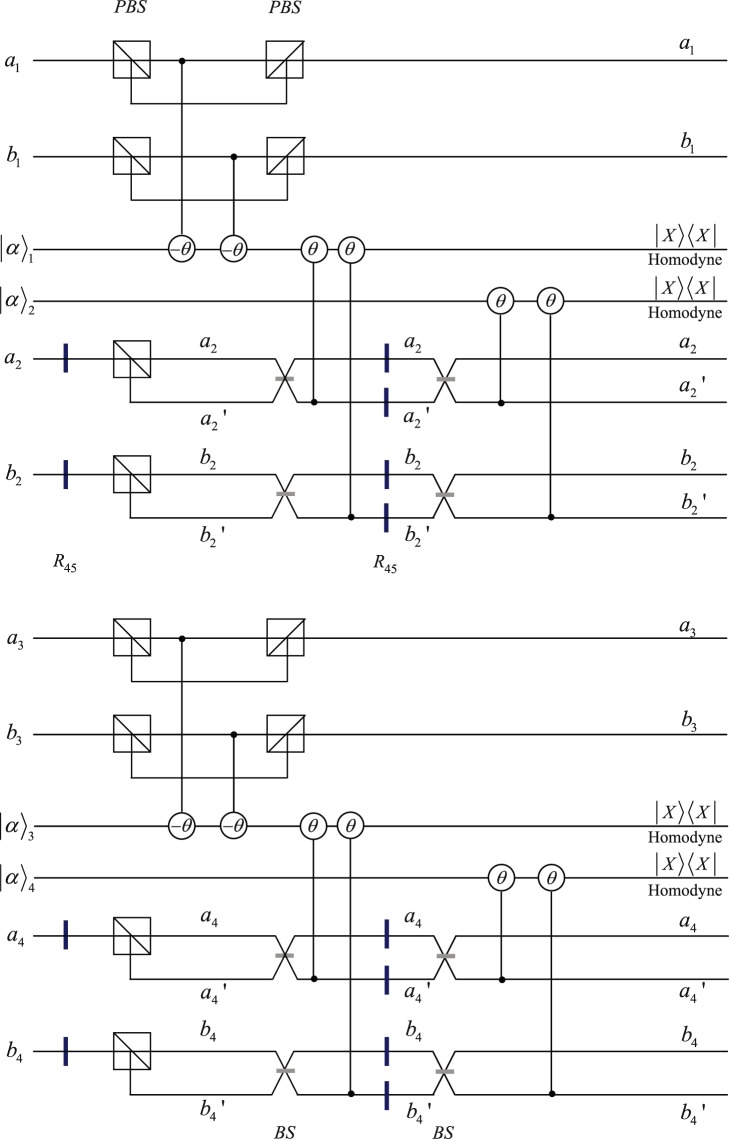
Quantum circuit for nonlocal implementation of CNOT operation in polarization DOF. Polarization Beam Splitter(PBS) transmits horizontal polarization and reflects vertical polarization. *θ* and −*θ* denote the cross-Kerr nonlinearities between the signal photons and the probe coherent beams. ${A}_{1},{B}_{1}$ are two spatial modes of photon ${A}_{1}$. ${A}_{2},{B}_{2}$, ${a^{\prime} }_{2},{b^{\prime} }_{2}$ represent four spatial modes of photon ${A}_{2}$, ${a}_{3},{b}_{3}$ are two spatial modes of photon ${B}_{2}$, ${a}_{4},{b}_{4},{a^{\prime} }_{4},{b^{\prime} }_{4}$ represent four spatial modes of photon ${B}_{1}$. Beam Splitter (BS) can implement Hadamard operation in spatial-mode DOF. The wave plate ${R}_{45}$ is used to implement Hadamard operation in polarization DOF.

The quantum circuit for nonlocal implementation of CNOT operation in polarization DOF is shown in Fig. 1. Here ±*θ* represent the cross-Kerr nonlinearity materia which add the phase shifts ${e}^{\pm i\theta }$ to the coherent probe states $|\alpha {\rangle }_{1}$, $|\alpha {\rangle }_{3}$ if the number of photon in the signal state coupled with the coherent probe beam is 1^68–71^. Polarizing beam splitters (PBS) can transmit horizontal polarization and reflect vertical polarization. The wave plate *R*_45_ is used to implement Hadamard operation on polarization DOF^72^

5 $$|H\rangle \to \frac{1}{\sqrt{2}}(|H\rangle +|V\rangle ),|V\rangle \to \frac{1}{\sqrt{2}}(|H\rangle -|V\rangle ).$$

Beam splitter(BS) can implement Hadamard operation on spatial-mode DOF

6 $$|{a}_{2}\rangle \to \frac{1}{\sqrt{2}}(|{a}_{2}\rangle +|{a^{\prime} }_{2}\rangle ),|{b}_{2}\rangle \to \frac{1}{\sqrt{2}}(|{b}_{2}\rangle +|{b^{\prime} }_{2}\rangle ).$$

To implement CNOT operation in polarization DOF nonlocally, Alice and Bob first let photons *A*_1_, *B*_2_ interact with probe coherent beams $|\alpha {\rangle }_{1}$, $|\alpha {\rangle }_{3}$ via cross-Kerr nonlinearities, then perform Hadamard operations on photons ${A}_{2},{B}_{1}$ in polarization DOF. Subsequently, Alice and Bob let photons ${A}_{2},{B}_{1}$ pass through PBSs in spatial modes ${a}_{2},{b}_{2},{a}_{4},{b}_{4}$ and let photons ${A}_{2},{B}_{1}$ interact with probe coherent beams $|\alpha {\rangle }_{1}$, $|\alpha {\rangle }_{3}$ via cross-Kerr nonlinearities. After the cross-Kerr interactions between photons ${A}_{2},{B}_{1}$ and probe coherent beams $|\alpha {\rangle }_{1}$, $|\alpha {\rangle }_{3}$, Alice and Bob perform X quadrature measurements on probe coherent beams $|\alpha {\rangle }_{1}$, $|\alpha {\rangle }_{3}$. After the X quadrature measurements, Alice and Bob apply Hadamard operations on photons ${A}_{2},{B}_{1}$ in polarization and spatial-mode DOFs, and pick up corresponding phase shifts via cross-Kerr nonlinearities between photons ${A}_{2},{B}_{1}$ and probe coherent beams $|\alpha {\rangle }_{2}$, $|\alpha {\rangle }_{4}$. The target state in polarization DOF can be prepared if the agents performing corresponding unitary operations on their photons according to the X quadrature measurement results of probe coherent beams $|\alpha {\rangle }_{1}$, $|\alpha {\rangle }_{3},|\alpha {\rangle }_{2}$, $|\alpha {\rangle }_{4}$.

After photons ${A}_{1},{B}_{2}$ interact with probe cohere beams $|\alpha {\rangle }_{1}$, $|\alpha {\rangle }_{3}$, the state of photons ${A}_{1},{A}_{2},{B}_{2},{B}_{1}$ evolves to^67,68^

7 $$\begin{array}{rcl}|{\Psi }_{2}\rangle  & = & ({\alpha }_{1}|H\rangle |\alpha {e}^{-i\theta }{\rangle }_{1}+{\beta }_{1}|V\rangle |\alpha {\rangle }_{1})\\  &  & \times \,(|HH\rangle |\alpha {e}^{-i\theta }{\rangle }_{3}+|VV\rangle |\alpha {\rangle }_{3})({\alpha }_{2}|H\rangle +{\beta }_{2}|V\rangle )\\  &  & \times \,({\gamma }_{1}|{a}_{1}\rangle +{\delta }_{1}|{b}_{1}\rangle )(|{a}_{2}{a}_{3}\rangle +|{b}_{2}{b}_{3}\rangle )\\  &  & \times \,({\gamma }_{2}|{a}_{4}\rangle +{\delta }_{2}|{b}_{4}\rangle ).\end{array}$$

To apply CNOT operation in polarization DOF nonlocally, Alice and Bob perform Hadamard operations on photons ${A}_{2},{B}_{1}$ via wave plates ${R}_{45}$. After the Hadamard operations, the state of photons ${A}_{1},{A}_{2},{B}_{2},{B}_{1}$ becomes

8 $$\begin{array}{rcl}|{\Psi }_{3}\rangle  & = & ({\alpha }_{1}|H\rangle |\alpha {e}^{-i\theta }{\rangle }_{1}+{\beta }_{1}|V\rangle |\alpha {\rangle }_{1})\\  &  & \times \,(|HH\rangle |\alpha {e}^{-i\theta }{\rangle }_{3}+|VH\rangle |\alpha {e}^{-i\theta }{\rangle }_{3}\\  &  & +\,|HV\rangle |\alpha {\rangle }_{3}-|VV\rangle |\alpha {\rangle }_{3})({\alpha ^{\prime} }_{2}|H\rangle +{\beta ^{\prime} }_{2}|V\rangle )\\  &  & \times \,({\gamma }_{1}|{a}_{1}\rangle +{\delta }_{1}|{b}_{1}\rangle )(|{a}_{2}{a}_{3}\rangle +|{b}_{2}{b}_{3}\rangle )\\  &  & \times \,({\gamma }_{2}|{a}_{4}\rangle +{\delta }_{2}|{b}_{4}\rangle ),\end{array}$$

where $${\alpha ^{\prime} }_{2}=\frac{1}{\sqrt{2}}({\alpha }_{2}+{\beta }_{2})$$, $${\beta ^{\prime} }_{2}=\frac{1}{\sqrt{2}}({\alpha }_{2}-{\beta }_{2})$$.

Subsequently, Alice and Bob let photons ${A}_{2},{B}_{1}$ pass through PBSs in spatial modes ${a}_{2},{b}_{2},{a}_{4},{b}_{4}$. The state of photons ${A}_{1},{A}_{2},{B}_{2},{B}_{1}$ becomes

9 $$\begin{array}{rcl}|{\Psi }_{4}\rangle  & = & ({\alpha }_{1}|H\rangle |\alpha {e}^{-i\theta }{\rangle }_{1}+{\beta }_{1}|V\rangle |\alpha {\rangle }_{1})\\  &  & \times \,(|HH\rangle |\alpha {e}^{-i\theta }{\rangle }_{3}|{a}_{2}{a}_{3}\rangle +|VH\rangle |\alpha {e}^{-i\theta }{\rangle }_{3}|{a^{\prime} }_{2}{a}_{3}\rangle \\  &  & +\,|HV\rangle |\alpha {\rangle }_{3}|{a}_{2}{a}_{3}\rangle -|VV\rangle |\alpha {\rangle }_{3}|{a^{\prime} }_{2}{a}_{3}\rangle \\  &  & +\,|HH\rangle |\alpha {e}^{-i\theta }{\rangle }_{3}|{b}_{2}{b}_{3}\rangle +|VH\rangle |\alpha {e}^{-i\theta }{\rangle }_{3}|{b^{\prime} }_{2}{b}_{3}\rangle \\  &  & +\,|HV\rangle |\alpha {\rangle }_{3}|{b}_{2}{b}_{3}\rangle -|VV\rangle |\alpha {\rangle }_{3}|{b^{\prime} }_{2}{b}_{3}\rangle )({\alpha ^{\prime} }_{2}{\gamma }_{2}|H{a}_{4}\rangle \\  &  & +\,{\alpha ^{\prime} }_{2}{\delta }_{2}|H{b}_{4}\rangle +{\beta ^{\prime} }_{2}{\gamma }_{2}|V{a^{\prime} }_{4}\rangle \\  &  & +\,{\beta ^{\prime} }_{2}{\delta }_{2}|V{b^{\prime} }_{4}\rangle )({\gamma }_{1}|{a}_{1}\rangle +{\delta }_{1}|{b}_{1}\rangle ),\end{array}$$

where ${a}_{2},{b}_{2},{a^{\prime} }_{2},{b^{\prime} }_{2}$ are four spatial modes of photon ${A}_{2}$ and ${a}_{4},{b}_{4},{a^{\prime} }_{4},{b^{\prime} }_{4}$ represent four spatial modes of photon ${B}_{1}$.

To implement Hadamard operation on photons ${A}_{2},{B}_{1}$ in spatial-mode DOF, Alice and Bob let photons ${A}_{2},{B}_{1}$ in spatial modes ${a}_{2},{b}_{2},{a^{\prime} }_{2},{b^{\prime} }_{2}$ and ${a}_{4},{b}_{4},{a^{\prime} }_{4},{b^{\prime} }_{4}$ pass through BSs, interact with probe coherent beams $|\alpha {\rangle }_{1}$, $|\alpha {\rangle }_{3}$ with the cross-Kerr linearities. The interaction between photon ${A}_{2}$ and $|\alpha {\rangle }_{1}$ adds a phase shift ${e}^{i\theta }$ to probe coherent beam if the number of photon in spatial mode ${a^{\prime} }_{2}$ or ${b^{\prime} }_{2}$ is 1. The interaction between photon ${B}_{2}$ and $|\alpha {\rangle }_{3}$ adds a phase shift ${e}^{i\theta }$ to probe coherent beam if the number of photon in spatial mode ${a^{\prime} }_{4}$ or ${b^{\prime} }_{4}$ is 1. After the cross-Kerr linearities, the state of four photons becomes (without normalization)

10 $$\begin{array}{rcl}|{\Psi }_{5}\rangle  & = & {\alpha }_{1}|H\rangle |\alpha {e}^{-i\theta }{\rangle }_{1}(|HH\rangle |\alpha {e}^{-i\theta }{\rangle }_{3}+|VH\rangle |\alpha {e}^{-i\theta }{\rangle }_{3}\\  &  & +\,|HV\rangle |\alpha {\rangle }_{3}-|VV\rangle |\alpha {\rangle }_{3})(|{a}_{2}{a}_{3}\rangle +|{b}_{2}{b}_{3}\rangle )\\  &  & \times \,({\alpha ^{\prime} }_{2}{\gamma }_{2}|H{a}_{4}\rangle +{\alpha ^{\prime} }_{2}{\delta }_{2}|H{b}_{4}\rangle +{\beta ^{\prime} }_{2}{\gamma }_{2}|V{a^{\prime} }_{4}\rangle \\  &  & +\,{\beta ^{\prime} }_{2}{\delta }_{2}|V{b^{\prime} }_{4}\rangle )({\gamma }_{1}|{a}_{1}\rangle +{\delta }_{1}|{b}_{1}\rangle )\\  &  & +\,{\alpha }_{1}|H\rangle |\alpha {e}^{-i\theta }{\rangle }_{1}(|HH\rangle |\alpha {\rangle }_{3}+|VH\rangle |\alpha {\rangle }_{3}\\  &  & +\,|HV\rangle |\alpha {e}^{i\theta }{\rangle }_{3}-|VV\rangle |\alpha {e}^{i\theta }{\rangle }_{3})\\  &  & \times \,(|{a}_{2}{a}_{3}\rangle +|{b}_{2}{b}_{3}\rangle )({\alpha ^{\prime} }_{2}{\gamma }_{2}|H{a}_{4}\rangle +{\alpha ^{\prime} }_{2}{\delta }_{2}|H{b}_{4}\rangle \\  &  & -\,{\beta ^{\prime} }_{2}{\gamma }_{2}|V{a^{\prime} }_{4}\rangle -{\beta ^{\prime} }_{2}{\delta }_{2}|V{b^{\prime} }_{4}\rangle )({\gamma }_{1}|{a}_{1}\rangle +{\delta }_{1}|{b}_{1}\rangle )\\  &  & +\,{\alpha }_{1}|H\rangle |\alpha {\rangle }_{1}(|HH\rangle |\alpha {e}^{-i\theta }{\rangle }_{3}-|VH\rangle |\alpha {e}^{-i\theta }{\rangle }_{3}\\  &  & +\,|HV\rangle |\alpha {\rangle }_{3}+|VV\rangle |\alpha {\rangle }_{3})(|{a^{\prime} }_{2}{a}_{3}\rangle +|{b^{\prime} }_{2}{b}_{3}\rangle )({\alpha ^{\prime} }_{2}{\gamma }_{2}|H{a}_{4}\rangle \\  &  & +\,{\alpha ^{\prime} }_{2}{\delta }_{2}|H{b}_{4}\rangle +{\beta ^{\prime} }_{2}{\gamma }_{2}|V{a^{\prime} }_{4}\rangle +{\beta ^{\prime} }_{2}{\delta }_{2}|V{b^{\prime} }_{4}\rangle )\\  &  & \times \,({\gamma }_{1}|{a}_{1}\rangle +{\delta }_{1}|{b}_{1}\rangle )+{\alpha }_{1}|H\rangle |\alpha {\rangle }_{1}(|HH\rangle |\alpha {\rangle }_{3}\\  &  & -\,|VH\rangle |\alpha {\rangle }_{3}+|HV\rangle |\alpha {e}^{i\theta }{\rangle }_{3}+|VV\rangle |\alpha {e}^{i\theta }{\rangle }_{3})\\  &  & \times \,(|{a^{\prime} }_{2}{a}_{3}\rangle +|{b^{\prime} }_{2}{b}_{3}\rangle )({\alpha ^{\prime} }_{2}{\gamma }_{2}|H{a}_{4}\rangle +{\alpha ^{\prime} }_{2}{\delta }_{2}|H{b}_{4}\rangle \\  &  & -\,{\beta ^{\prime} }_{2}{\gamma }_{2}|V{a^{\prime} }_{4}\rangle -{\beta ^{\prime} }_{2}{\delta }_{2}|V{b^{\prime} }_{4}\rangle )({\gamma }_{1}|{a}_{1}\rangle +{\delta }_{1}|{b}_{1}\rangle )\\  &  & +\,{\beta }_{1}|V\rangle |\alpha {\rangle }_{1}(|HH\rangle |\alpha {e}^{-i\theta }{\rangle }_{3}+|VH\rangle |\alpha {e}^{-i\theta }{\rangle }_{3}\\  &  & +\,|HV\rangle |\alpha {\rangle }_{3}-|VV\rangle |\alpha {\rangle }_{3})(|{a}_{2}{a}_{3}\rangle +|{b}_{2}{b}_{3}\rangle )\\  &  & \times \,({\alpha ^{\prime} }_{2}{\gamma }_{2}|H{a}_{4}\rangle +{\alpha ^{\prime} }_{2}{\delta }_{2}|H{b}_{4}\rangle +{\beta ^{\prime} }_{2}{\gamma }_{2}|V{a^{\prime} }_{4}\rangle \\  &  & +\,{\beta ^{\prime} }_{2}{\delta }_{2}|V{b^{\prime} }_{4}\rangle )({\gamma }_{1}|{a}_{1}\rangle +{\delta }_{1}|{b}_{1}\rangle )+{\beta }_{1}|V\rangle |\alpha {\rangle }_{1}\\  &  & \times \,(|HH\rangle |\alpha {\rangle }_{3}+|VH\rangle |\alpha {\rangle }_{3}+|HV\rangle |\alpha {e}^{i\theta }{\rangle }_{3}\\  &  & -\,|VV\rangle |\alpha {e}^{i\theta }{\rangle }_{3})(|{a}_{2}{a}_{3}\rangle +|{b}_{2}{b}_{3}\rangle )({\alpha ^{\prime} }_{2}{\gamma }_{2}|H{a}_{4}\rangle \\  &  & +\,{\alpha ^{\prime} }_{2}{\delta }_{2}|H{b}_{4}\rangle -{\beta ^{\prime} }_{2}{\gamma }_{2}|V{a^{\prime} }_{4}\rangle -{\beta ^{\prime} }_{2}{\delta }_{2}|V{b^{\prime} }_{4}\rangle )\\  &  & \times \,({\gamma }_{1}|{a}_{1}\rangle +{\delta }_{1}|{b}_{1}\rangle )+{\beta }_{1}|V\rangle |\alpha {e}^{i\theta }{\rangle }_{1}\\  &  & \times \,(|HH\rangle |\alpha {e}^{-i\theta }{\rangle }_{3}-|VH\rangle |\alpha {e}^{-i\theta }{\rangle }_{3}\\  &  & +\,|HV\rangle |\alpha {\rangle }_{3}+|VV\rangle |\alpha {\rangle }_{3})(|{a^{\prime} }_{2}{a}_{3}\rangle \\  &  & +\,|{b^{\prime} }_{2}{b}_{3}\rangle )({\alpha ^{\prime} }_{2}{\gamma }_{2}|H{a}_{4}\rangle +{\alpha ^{\prime} }_{2}{\delta }_{2}|H{b}_{4}\rangle \\  &  & +\,{\beta ^{\prime} }_{2}{\gamma }_{2}|V{a^{\prime} }_{4}\rangle +{\beta ^{\prime} }_{2}{\delta }_{2}|V{b^{\prime} }_{4}\rangle )({\gamma }_{1}|{a}_{1}\rangle \\  &  & +\,{\delta }_{1}|{b}_{1}\rangle )+{\beta }_{1}|V\rangle |\alpha {e}^{i\theta }{\rangle }_{1}(|HH\rangle |\alpha {\rangle }_{3}-|VH\rangle |\alpha {\rangle }_{3}\\  &  & +\,|HV\rangle |\alpha {e}^{i\theta }{\rangle }_{3}+|VV\rangle |\alpha {e}^{i\theta }{\rangle }_{3})(|{a^{\prime} }_{2}{a}_{3}\rangle +|{b^{\prime} }_{2}{b}_{3}\rangle )\\  &  & \times \,({\alpha ^{\prime} }_{2}{\gamma }_{2}|H{a}_{4}\rangle +{\alpha ^{\prime} }_{2}{\delta }_{2}|H{b}_{4}\rangle -{\beta ^{\prime} }_{2}{\gamma }_{2}|V{a^{\prime} }_{4}\rangle \\  &  & -\,{\beta ^{\prime} }_{2}{\delta }_{2}|V{b^{\prime} }_{4}\rangle )({\gamma }_{1}|{a}_{1}\rangle +{\delta }_{1}|{b}_{1}\rangle ).\end{array}$$

Since the X quadrature measurements performed on probe coherent beams $|\alpha {\rangle }_{1}$, $|\alpha {\rangle }_{3}$ can distinguish $|\alpha {e}^{\pm i\theta }{\rangle }_{1}$, $|\alpha {e}^{\pm i\theta }{\rangle }_{3}$ from $|\alpha {\rangle }_{1}$, $|\alpha {\rangle }_{3}$, the measurement results of the two X quadrature measurements are $|\alpha {e}^{\pm i\theta }{\rangle }_{1}|\alpha {e}^{\pm i\theta }{\rangle }_{3}$, $|\alpha {e}^{\pm i\theta }{\rangle }_{1}|\alpha {\rangle }_{3}$, $|\alpha {\rangle }_{1}|\alpha {e}^{\pm i\theta }{\rangle }_{3}$ or $|\alpha {\rangle }_{1}|\alpha {\rangle }_{3}$.

Suppose the measurement results of the two X quadrature measurements are $|\alpha {e}^{\pm i\theta }{\rangle }_{1}|\alpha {e}^{\pm i\theta }{\rangle }_{3}$, the state of photons ${A}_{1},{A}_{2},{B}_{2},{B}_{1}$ evolves to

11 $$\begin{array}{rcl}|{\psi }_{1}\rangle  & = & [{\alpha }_{1}|H\rangle (|HH\rangle |{a}_{2}{a}_{3}\rangle +|VH\rangle |{a}_{2}{a}_{3}\rangle +|HH\rangle |{b}_{2}{b}_{3}\rangle \\  &  & +\,|VH\rangle |{b}_{2}{b}_{3}\rangle )({\alpha ^{\prime} }_{2}{\gamma }_{2}|H{a}_{4}\rangle +{\alpha ^{\prime} }_{2}{\delta }_{2}|H{b}_{4}\rangle +{\beta ^{\prime} }_{2}{\gamma }_{2}\\  &  & \times \,|V{a}_{4}\rangle +{\beta ^{\prime} }_{2}{\delta }_{2}|V{b}_{4}\rangle )+{\alpha }_{1}|H\rangle (|HV\rangle |{a}_{2}{a}_{3}\rangle \\  &  & -\,|VV\rangle |{a}_{2}{a}_{3}\rangle +|HV\rangle |{b}_{2}{b}_{3}\rangle -|VV\rangle |{b}_{2}{b}_{3}\rangle )({\alpha ^{\prime} }_{2}{\gamma }_{2}|H{a^{\prime} }_{4}\rangle \\  &  & +\,{\alpha ^{\prime} }_{2}{\delta }_{2}|H{b^{\prime} }_{4}\rangle -{\beta ^{\prime} }_{2}{\gamma }_{2}|V{a^{\prime} }_{4}\rangle -{\beta ^{\prime} }_{2}{\delta }_{2}|V{b^{\prime} }_{4}\rangle )\\  &  & +\,{\beta }_{1}|V\rangle (|HH\rangle |{a^{\prime} }_{2}{a}_{3}\rangle -|VH\rangle |{a^{\prime} }_{2}{a}_{3}\rangle \\  &  & +\,|HH\rangle |{b^{\prime} }_{2}{b}_{3}\rangle -|VH\rangle |{b^{\prime} }_{2}{b}_{3}\rangle )({\alpha ^{\prime} }_{2}{\gamma }_{2}|H{a}_{4}\rangle \\  &  & +\,{\alpha ^{\prime} }_{2}{\delta }_{2}|H{b}_{4}\rangle +{\beta ^{\prime} }_{2}{\gamma }_{2}|V{a}_{4}\rangle +{\beta ^{\prime} }_{2}{\delta }_{2}|V{b}_{4}\rangle )\\  &  & +\,{\beta }_{1}|V\rangle (|HV\rangle |{a^{\prime} }_{2}{a}_{3}\rangle +|VV\rangle |{a^{\prime} }_{2}{a}_{3}\rangle \\  &  & +\,|HV\rangle |{b^{\prime} }_{2}{b}_{3}\rangle +|VV\rangle |{b^{\prime} }_{2}{b}_{3}\rangle )({\alpha ^{\prime} }_{2}{\gamma }_{2}|H{a^{\prime} }_{4}\rangle \\  &  & +\,{\alpha ^{\prime} }_{2}{\delta }_{2}|H{b^{\prime} }_{4}\rangle -{\beta ^{\prime} }_{2}{\gamma }_{2}|V{a^{\prime} }_{4}\rangle \\  &  & -\,{\beta ^{\prime} }_{2}{\delta }_{2}|V{b^{\prime} }_{4}\rangle )]({\gamma }_{1}|{a}_{1}\rangle +{\delta }_{1}|{b}_{1}\rangle ).\end{array}$$

For implementation of CNOT operation in polarization DOF nonlocally, Alice and Bob apply Hadamard operations on photons ${A}_{2},{B}_{1}$ in polarization and spatial-mode DOFs. After the Hadamard operations, the state of four photons becomes (without normalization)

12 $$\begin{array}{rcl}|{\psi }_{2}\rangle  & = & [{\alpha }_{1}|H\rangle |HH\rangle (|{a}_{2}{a}_{3}\rangle +|{a^{\prime} }_{2}{a}_{3}\rangle +|{b}_{2}{b}_{3}\rangle +|{b^{\prime} }_{2}{b}_{3}\rangle )\\  &  & \times \,({\alpha }_{2}|H\rangle +{\beta }_{2}|V\rangle )({\gamma }_{2}|{a}_{4}\rangle +{\delta }_{2}|{b}_{4}\rangle +{\gamma }_{2}|{a^{\prime} }_{4}\rangle +{\delta }_{2}|{b^{\prime} }_{4}\rangle )\\  &  & +\,{\alpha }_{1}|H\rangle |VV\rangle (|{a}_{2}{a}_{3}\rangle +|{a^{\prime} }_{2}{a}_{3}\rangle +|{b}_{2}{b}_{3}\rangle +|{b^{\prime} }_{2}{b}_{3}\rangle )\\  &  & \times \,({\beta }_{2}|H\rangle +{\alpha }_{2}|V\rangle )({\gamma }_{2}|{a}_{4}\rangle +{\delta }_{2}|{b}_{4}\rangle -{\gamma }_{2}|{a^{\prime} }_{4}\rangle -{\delta }_{2}|{b^{\prime} }_{4}\rangle )\\  &  & +\,{\beta }_{1}|V\rangle |VH\rangle (|{a}_{2}{a}_{3}\rangle -|{a^{\prime} }_{2}{a}_{3}\rangle +|{b}_{2}{b}_{3}\rangle -|{b^{\prime} }_{2}{b}_{3}\rangle )\\  &  & \times \,({\alpha }_{2}|H\rangle +{\beta }_{2}|V\rangle )({\gamma }_{2}|{a}_{4}\rangle +{\delta }_{2}|{b}_{4}\rangle +{\gamma }_{2}|{a^{\prime} }_{4}\rangle +{\delta }_{2}|{b^{\prime} }_{4}\rangle )\\  &  & +\,{\beta }_{1}|V\rangle |HV\rangle (|{a}_{2}{a}_{3}\rangle -|{a^{\prime} }_{2}{a}_{3}\rangle +|{b}_{2}{b}_{3}\rangle -|{b^{\prime} }_{2}{b}_{3}\rangle )\\  &  & \times \,({\beta }_{2}|H\rangle +{\alpha }_{2}|V\rangle )({\gamma }_{2}|{a}_{4}\rangle +{\delta }_{2}|{b}_{4}\rangle -{\gamma }_{2}|{a^{\prime} }_{4}\rangle -{\delta }_{2}|{b^{\prime} }_{4}\rangle )]\\  &  & \times \,({\gamma }_{1}|{a}_{1}\rangle +{\delta }_{1}|{b}_{1}\rangle ).\end{array}$$

To implement the nonlocal CNOT operation in polarization DOF, Alice and Bob let photons ${A}_{2},{B}_{1}$ interact with probe coherent beams $|\alpha {\rangle }_{2}$, $|\alpha {\rangle }_{4}$ via cross-Kerr nonlinearities and perform X quadrature measurements on probe coherent beams. The interaction between photon ${A}_{2}$ and $|\alpha {\rangle }_{2}$ adds a phase shift ${e}^{i\theta }$ to probe coherent beam if the number of photon in spatial mode ${a^{\prime} }_{2}$ or ${b^{\prime} }_{2}$ is 1.

The relation between the X quadrature measurements results of probe coherent beams $|\alpha {\rangle }_{2}$, $|\alpha {\rangle }_{4}$, the state of four photons after the X quadrature measurements and the recovery operation performed by Alice and Bob according to the measurement results is shown in Table 1. Here

13 $$\begin{array}{rcl}|{\psi }_{3}\rangle  & = & [{\alpha }_{1}|H\rangle |HH\rangle ({\alpha }_{2}|H\rangle +{\beta }_{2}|V\rangle )+{\alpha }_{1}|H\rangle |VV\rangle \\  &  & \times \,({\beta }_{2}|H\rangle +{\alpha }_{2}|V\rangle )+{\beta }_{1}|V\rangle |VH\rangle \\  &  & \times \,({\alpha }_{2}|H\rangle +{\beta }_{2}|V\rangle )+{\beta }_{1}|V\rangle |HV\rangle \\  &  & \times \,({\beta }_{2}|H\rangle +{\alpha }_{2}|V\rangle )]({\gamma }_{1}|{a}_{1}\rangle +{\delta }_{1}|{b}_{1}\rangle )\\  &  & \times \,(|{a}_{2}{a}_{3}\rangle +|{b}_{2}{b}_{3}\rangle )({\gamma }_{2}|{a}_{4}\rangle +{\delta }_{2}|{b}_{4}\rangle ),\end{array}$$

**Table 1:** The relation between the X quadrature measurements results of probe coherent beams $|\alpha {\rangle }_{2}$, $|\alpha {\rangle }_{4}$, the state of four photons after the X quadrature measurements and unitaty operation with which Alice and Bob can transform state of photons ${A}_{1},{A}_{2},{B}_{2},{B}_{1}$ to target state $|{\psi }_{3}\rangle$ in polarization DOF in accordance with the X quadrature measurement results.

*R*_2_	*R*_4_	State	*U*_*t*_
$|\alpha {\rangle }_{2}$	$|\alpha {\rangle }_{4}$	$|{\psi }_{3}\rangle$	*I*
$|\alpha {\rangle }_{2}$	$|\alpha {e}^{i\theta }{\rangle }_{4}$	${({\sigma }_{x}^{s})}_{{a^{\prime} }_{4}{a}_{4}}{({\sigma }_{x}^{s})}_{{b^{\prime} }_{4}{b}_{4}}{({\sigma }_{z}^{p})}_{{B}_{2}}|{\psi }_{3}\rangle$	${({\sigma }_{x}^{s})}_{{a^{\prime} }_{4}{a}_{4}}{({\sigma }_{x}^{s})}_{{b^{\prime} }_{4}{b}_{4}}{({\sigma }_{z}^{p})}_{{B}_{2}}$
$|\alpha {e}^{i\theta }{\rangle }_{2}$	$|\alpha {\rangle }_{4}$	${({\sigma }_{x}^{s})}_{{a^{\prime} }_{2}{a}_{2}}{({\sigma }_{x}^{s})}_{{b^{\prime} }_{2}{b}_{2}}{({\sigma }_{z}^{p})}_{{A}_{1}}|{\psi }_{3}\rangle$	${({\sigma }_{x}^{s})}_{{a^{\prime} }_{2}{a}_{2}}{({\sigma }_{x}^{s})}_{{b^{\prime} }_{2}{b}_{2}}{({\sigma }_{z}^{p})}_{{A}_{1}}$
$|\alpha {e}^{i\theta }{\rangle }_{2}$	$|\alpha {e}^{i\theta }{\rangle }_{4}$	${({\sigma }_{x}^{s})}_{{a^{\prime} }_{2}{a}_{2}}{({\sigma }_{x}^{s})}_{{b^{\prime} }_{2}{b}_{2}}{({\sigma }_{x}^{s})}_{{a^{\prime} }_{4}{a}_{4}}{({\sigma }_{x}^{s})}_{{b^{\prime} }_{4}{b}_{4}}{({\sigma }_{z}^{p})}_{{A}_{2}}|{\psi }_{3}\rangle$	${({\sigma }_{x}^{s})}_{{a^{\prime} }_{2}{a}_{2}}{({\sigma }_{x}^{s})}_{{b^{\prime} }_{2}{b}_{2}}{({\sigma }_{x}^{s})}_{{a^{\prime} }_{4}{a}_{4}}{({\sigma }_{x}^{s})}_{{b^{\prime} }_{4}{b}_{4}}{({\sigma }_{z}^{p})}_{{A}_{2}}$

*R*_2_, *R*_4_ represent the X quadrature measurements results of probe coherent beams $|\alpha {\rangle }_{2}$, $|\alpha {\rangle }_{4}$.

${({\sigma }_{x}^{s})}_{{f}_{j}{f^{\prime} }_{j}}=|{f}_{j}\rangle \langle {f^{\prime} }_{j}|+|{f^{\prime} }_{j}\rangle \langle {f}_{j}|$ $({f}_{j}={a}_{2},{b}_{2},{a}_{4},{b}_{4})$ represent the bit-flip operations in spatial modes ${f}_{j},{f^{\prime} }_{j}$. ${({\sigma }_{z}^{p})}_{i}=|H\rangle \langle H|-|V\rangle \langle V|$, $i={B}_{2},{A}_{1},{A}_{2}$ represents ${\sigma }_{z}^{p}$ operation in the polarization DOF of photon i^72^. For example, we assume the outcome of the X quadrature measurements is $|\alpha {\rangle }_{2}|\alpha {e}^{i\theta }{\rangle }_{4}$. In this situation, the state of photons becomes

14 $$\begin{array}{rcl}|{\psi ^{\prime} }_{3}\rangle  & = & [{\alpha }_{1}|H\rangle |HH\rangle ({\alpha }_{2}|H\rangle +{\beta }_{2}|V\rangle )-{\alpha }_{1}|H\rangle |VV\rangle \\  &  & \times \,({\beta }_{2}|H\rangle +{\alpha }_{2}|V\rangle )+{\beta }_{1}|V\rangle |VH\rangle \\  &  & \times \,({\alpha }_{2}|H\rangle +{\beta }_{2}|V\rangle )-{\beta }_{1}|V\rangle |HV\rangle \\  &  & \times \,({\beta }_{2}|H\rangle +{\alpha }_{2}|V\rangle )]({\gamma }_{1}|{a}_{1}\rangle +{\delta }_{1}|{b}_{1}\rangle )\\  &  & \times \,(|{a}_{2}{a}_{3}\rangle +|{b}_{2}{b}_{3}\rangle )({\gamma }_{2}|{a^{\prime} }_{4}\rangle +{\delta }_{2}|{b^{\prime} }_{4}\rangle ).\end{array}$$

Bob can transform $|{\psi ^{\prime} }_{3}\rangle$ to the target state $|{\psi }_{3}\rangle$ by performing unitary operation operation ${({\sigma }_{x}^{s})}_{{a^{\prime} }_{4}{a}_{4}}{({\sigma }_{x}^{s})}_{{b^{\prime} }_{4}{b}_{4}}{({\sigma }_{z}^{p})}_{{B}_{2}}$.

For the other case, the relation between the X quadrature measurements results of probe coherent beams $|\alpha {\rangle }_{1}$, $|\alpha {\rangle }_{3}$, the state of four photons and the recovery operation performed by Alice and Bob according to the measurement results is shown in Table 2. Here ${({\sigma }_{x}^{p})}_{{B}_{2}}=|H\rangle \langle V|+|V\rangle \langle H|$ represents ${\sigma }_{x}^{p}$ operation in the polarization DOF of photon ${B}_{2}$. For example, we also assume the X quadrature measurements result is $|\alpha {\rangle }_{1}|\alpha {e}^{\pm i\theta }{\rangle }_{3}$. In this situation, the state of photons evolves to

15 $$\begin{array}{rcl}|{\psi ^{\prime} }_{1}\rangle  & = & [{\alpha }_{1}|H\rangle (|HH\rangle |{a^{\prime} }_{2}{a}_{3}\rangle -|VH\rangle |{a^{\prime} }_{2}{a}_{3}\rangle \\  &  & +\,|HH\rangle |{b^{\prime} }_{2}{b}_{3}\rangle -|VH\rangle |{b^{\prime} }_{2}{b}_{3}\rangle )({\alpha ^{\prime} }_{2}{\gamma }_{2}|H{a}_{4}\rangle \\  &  & +\,{\alpha ^{\prime} }_{2}{\delta }_{2}|H{b}_{4}\rangle +{\beta ^{\prime} }_{2}{\gamma }_{2}|V{a}_{4}\rangle +{\beta ^{\prime} }_{2}{\delta }_{2}|V{b}_{4}\rangle )\\  &  & +\,{\alpha }_{1}|H\rangle (|HV\rangle |{a^{\prime} }_{2}{a}_{3}\rangle +|VV\rangle |{a^{\prime} }_{2}{a}_{3}\rangle \\  &  & +\,|HV\rangle |{b^{\prime} }_{2}{b}_{3}\rangle +|VV\rangle |{b^{\prime} }_{2}{b}_{3}\rangle )({\alpha ^{\prime} }_{2}{\gamma }_{2}|H{a^{\prime} }_{4}\rangle \\  &  & +\,{\alpha ^{\prime} }_{2}{\delta }_{2}|H{b^{\prime} }_{4}\rangle -{\beta ^{\prime} }_{2}{\gamma }_{2}|V{a^{\prime} }_{4}\rangle -{\beta ^{\prime} }_{2}{\delta }_{2}|V{b^{\prime} }_{4}\rangle )\\  &  & +\,{\beta }_{1}|V\rangle (|HH\rangle |{a}_{2}{a}_{3}\rangle +|VH\rangle |{a}_{2}{a}_{3}\rangle \\  &  & +\,|HH\rangle |{b}_{2}{b}_{3}\rangle +|VH\rangle |{b}_{2}{b}_{3}\rangle )({\alpha ^{\prime} }_{2}{\gamma }_{2}|H{a}_{4}\rangle \\  &  & +\,{\alpha ^{\prime} }_{2}{\delta }_{2}|H{b}_{4}\rangle +{\beta ^{\prime} }_{2}{\gamma }_{2}|V{a}_{4}\rangle +{\beta ^{\prime} }_{2}{\delta }_{2}|V{b}_{4}\rangle )\\  &  & +\,{\beta }_{1}|V\rangle (|HV\rangle |{a}_{2}{a}_{3}\rangle -|VV\rangle |{a}_{2}{a}_{3}\rangle \\  &  & +\,|HV\rangle |{b}_{2}{b}_{3}\rangle -|VV\rangle |{b}_{2}{b}_{3}\rangle )({\alpha ^{\prime} }_{2}{\gamma }_{2}|H{a^{\prime} }_{4}\rangle +{\alpha ^{\prime} }_{2}{\delta }_{2}|H{b^{\prime} }_{4}\rangle \\  &  & -\,{\beta ^{\prime} }_{2}{\gamma }_{2}|V{a^{\prime} }_{4}\rangle -{\beta ^{\prime} }_{2}{\delta }_{2}|V{b^{\prime} }_{4}\rangle )]({\gamma }_{1}|{a}_{1}\rangle +{\delta }_{1}|{b}_{1}\rangle ).\end{array}$$

**Table 2:** The relation between the X quadrature measurements results of probe coherent beams $|\alpha {\rangle }_{1}$, $|\alpha {\rangle }_{3}$, the state of four photons after the X quadrature measurements and corresponding operation with which Alice and Bob can transform state of photons ${A}_{1},{A}_{2},{B}_{2},{B}_{1}$ to $|{\psi }_{1}\rangle$ in accordance with the X quadrature measurement results.

*R*_1_	*R*_3_	State	*U*_*t*_
$|\alpha {e}^{\pm i\theta }{\rangle }_{1}$	$|\alpha {e}^{\pm i\theta }{\rangle }_{3}$	$|{\psi }_{1}\rangle$	*I*
$|\alpha {e}^{\pm i\theta }{\rangle }_{1}$	$|\alpha {\rangle }_{3}$	${({\sigma }_{x}^{p})}_{{B}_{2}}{({\sigma }_{z}^{p})}_{{A}_{2}}|{\psi }_{1}\rangle$	${({\sigma }_{x}^{p})}_{{B}_{2}}{({\sigma }_{z}^{p})}_{{A}_{2}}$
$|\alpha {\rangle }_{1}$	$|\alpha {e}^{\pm i\theta }{\rangle }_{3}$	${({\sigma }_{x}^{s})}_{{a^{\prime} }_{2}{a}_{2}}{({\sigma }_{x}^{s})}_{{b^{\prime} }_{2}{b}_{2}}{({\sigma }_{z}^{p})}_{{A}_{2}}|{\psi }_{1}\rangle$	${({\sigma }_{x}^{s})}_{{a^{\prime} }_{2}{a}_{2}}{({\sigma }_{x}^{s})}_{{b^{\prime} }_{2}{b}_{2}}{({\sigma }_{z}^{p})}_{{A}_{2}}$
$|\alpha {\rangle }_{1}$	$|\alpha {\rangle }_{3}$	${({\sigma }_{x}^{s})}_{{a^{\prime} }_{2}{a}_{2}}{({\sigma }_{x}^{s})}_{{b^{\prime} }_{2}{b}_{2}}{({\sigma }_{x}^{p})}_{{B}_{2}}|{\psi }_{1}\rangle$	${({\sigma }_{x}^{s})}_{{a^{\prime} }_{2}{a}_{2}}{({\sigma }_{x}^{s})}_{{b^{\prime} }_{2}{b}_{2}}{({\sigma }_{x}^{p})}_{{B}_{2}}$

*R*_1_, *R*_3_ are the the X quadrature measurements results of probe coherent beams $|\alpha {\rangle }_{1}$, $|\alpha {\rangle }_{3}$.

Alice and Bob can transform $|{\psi ^{\prime} }_{1}\rangle$ to target state $|{\psi }_{1}\rangle$ by applying unitary operation ${({\sigma }_{x}^{s})}_{{a^{\prime} }_{2}{a}_{2}}{({\sigma }_{x}^{s})}_{{b^{\prime} }_{2}{b}_{2}}{({\sigma }_{z}^{p})}_{{A}_{2}}$.

#### Nonlocal CNOT operation in spatial-mode DOF

Now, let us consider the implementation of nonlocal CNOT operation in spatial-mode DOF. Assisted by spatial-mode entanglement of the hyperentangled state, cross-Kerr nonlinearity and linear-optics elements, CNOT operation in spatial-mode DOF can be implemented nonlocally.

To nonlocally implement CNOT operation in polarization and spatial-mode DOFs simultaneously, the agents implement nonlocal CNOT operation in spatial-mode DOF via spatial-mode entanglement of the hyperentangled state by using the spatial-mode state of photon ${A}_{1}$ as the control qubit after transform the state of photons ${A}_{1},{A}_{2},{B}_{2},{B}_{1}$ to $|{\psi }_{3}\rangle$.

**Figure 2:**
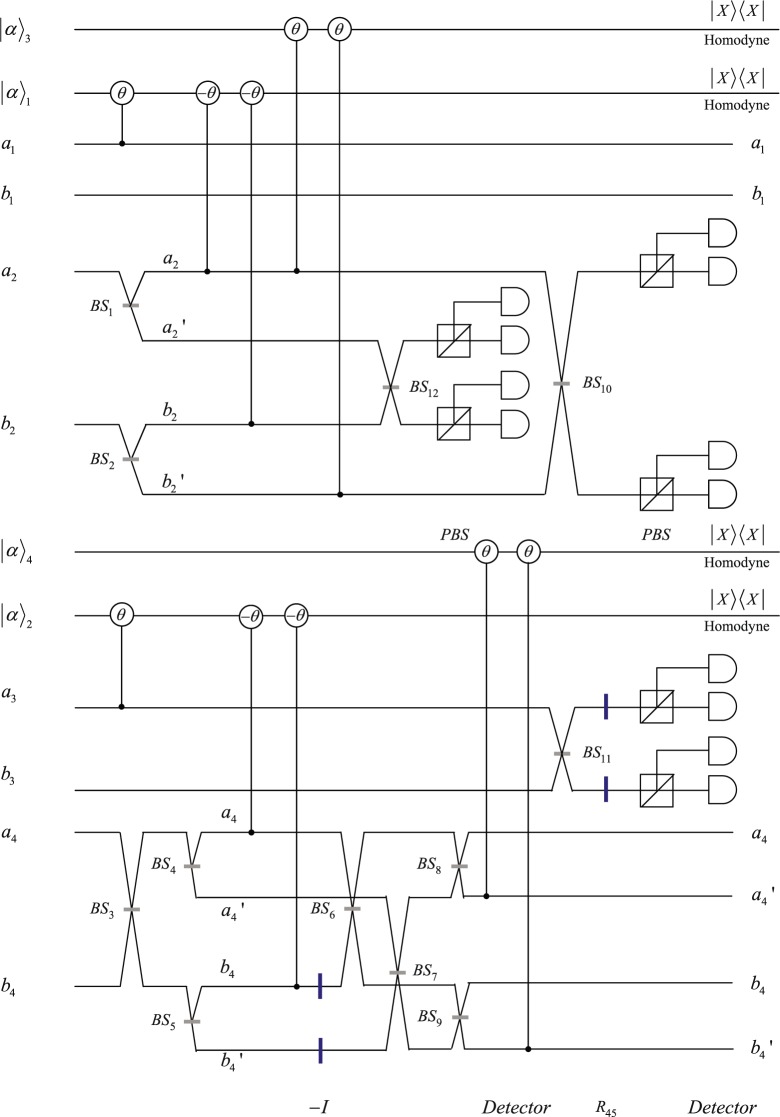
Quantum circuit for nonlocal implementation of CNOT operation in polarization DOF. Polarization Beam Splitter(PBS) transmit horizontal polarization and reflect vertical polarization. *θ* and −*θ* denote the cross-Kerr nonlinearities between the signal photons and the probe coherent beams. ${a}_{1},{b}_{1}$ are two spatial modes of photon ${A}_{1}$, ${a}_{2},{b}_{2},{a^{\prime} }_{2},{b^{\prime} }_{2}$ represent four spatial modes of photon ${A}_{2}$, ${a}_{3},{b}_{3}$ are two spatial modes of photon ${B}_{2}$, ${a}_{4},{b}_{4},{a^{\prime} }_{4},{b^{\prime} }_{4}$ represent four spatial modes of photon ${B}_{1}$. Beam Splitter (BS) can implement Hadamard operation in spatial-mode DOF. The wave plate ${R}_{45}$ is used to implement Hadamard operation in polarization DOF.

The quantum circuit for nonlocal implement of CNOT operation in spatial-mode DOF is shown in Fig. 2. Similar to ref.^67^, $(\,-\,I)=-\,|H\rangle \langle H|-|V\rangle \langle V|$ denotes the phase operation in photon ${B}_{1}$’s spatial modes ${b}_{4}$ and ${b^{\prime} }_{4}$. After photons ${A}_{2},{B}_{1}$ pass through BSs, Alice(Bob) first let photons ${A}_{1},{A}_{2}$ $({B}_{2},{B}_{1})$ interact with probe coherent beams $|\alpha {\rangle }_{1}$ ($|\alpha {\rangle }_{2}$) via cross-Kerr nonlinearity, then perform X quadrature measurement on the probe coherent beam. Alice and Bob apply corresponding unitary operations on photons ${A}_{2},{B}_{1}$ according to the measurement results, let photons ${A}_{2},{B}_{1}$ interact with probe coherent beams $|\alpha {\rangle }_{3}$, $|\alpha {\rangle }_{4}$. The hyper-parallel nonlocal CNOT operation in polarization and spatial-mode DOFs can be implemented if the agents perform corresponding unitary operations on photons ${A}_{1},{B}_{1}$ in accordance with the single-qubit measurement results of photons ${A}_{2},{B}_{2}$.

For implementation of nonlocal CNOT operation in spatial-mode DOF, Alice and Bob let photons ${A}_{2}$, ${B}_{1}$ pass through BSs which implement Hadamard operations in spatial modes ${a}_{2},{b}_{2},{a}_{4}$ and *b*_4_^72^

16 $$\begin{array}{ll}|{a}_{2}\rangle \to \frac{1}{\sqrt{2}}(|{a}_{2}\rangle +|{a^{\prime} }_{2}\rangle ), & |{b}_{2}\rangle \to \frac{1}{\sqrt{2}}(|{b}_{2}\rangle +|{b^{\prime} }_{2}\rangle ),\\ |{a}_{4}\rangle \to \frac{1}{\sqrt{2}}(|{a}_{4}\rangle +|{b}_{4}\rangle ), & |{b}_{4}\rangle \to \frac{1}{\sqrt{2}}(|{a}_{4}\rangle -|{b}_{4}\rangle ).\end{array}$$

Here ${a}_{2},{b}_{2},{a^{\prime} }_{2},{b^{\prime} }_{2}$ represent four spatial modes of photon ${A}_{2}$, ${a}_{4},{b}_{4}$ are two spatial modes of photon ${B}_{1}$.

The state of photons ${A}_{1},{A}_{2},{B}_{2},{B}_{1}$ is transformed from $|{\psi }_{3}\rangle$ to $|{\varphi }_{1}\rangle$ after photons ${A}_{2}$, ${B}_{1}$ pass through $B{S}_{j}(j=1,2,3)$ (without normalization). Here

17 $$\begin{array}{rcl}|{\varphi }_{1}\rangle  & = & |{\varphi }_{1}^{p}\rangle ({\gamma }_{1}|{a}_{1}\rangle +{\delta }_{1}|{b}_{1}\rangle )(|{a}_{2}{a}_{3}\rangle +|{a^{\prime} }_{2}{a}_{3}\rangle \\  &  & +\,|{b}_{2}{b}_{3}\rangle +|{b^{\prime} }_{2}{b}_{3}\rangle )({\gamma ^{\prime} }_{2}|{a}_{4}\rangle +{\delta ^{\prime} }_{2}|{b}_{4}\rangle ),\end{array}$$

${\gamma ^{\prime} }_{2}=\frac{1}{\sqrt{2}}({\gamma }_{2}+{\delta }_{2})$, ${\delta ^{\prime} }_{2}=\frac{1}{\sqrt{2}}({\gamma }_{2}-{\delta }_{2})$. $|{\varphi }_{1}^{p}\rangle$ represents the polarization state of photons ${A}_{1},{A}_{2},{B}_{2},{B}_{1}$

18 $$\begin{array}{rcl}|{\varphi }_{1}^{p}\rangle  & = & {\alpha }_{1}|H\rangle |HH\rangle ({\alpha }_{2}|H\rangle +{\beta }_{2}|V\rangle )+{\alpha }_{1}|H\rangle |VV\rangle \\  &  & \times \,({\beta }_{2}|H\rangle +{\alpha }_{2}|V\rangle )+{\beta }_{1}|V\rangle |VH\rangle \\  &  & \times \,({\alpha }_{2}|H\rangle +{\beta }_{2}|V\rangle )+{\beta }_{1}|V\rangle |HV\rangle \\  &  & \times \,({\beta }_{2}|H\rangle +{\alpha }_{2}|V\rangle ).\end{array}$$

To implement CNOT operation in spatial-mode DOF nonlocally, Bob let photon ${B}_{1}$ pass through $B{S}_{k},(k=4,5)$ which implement Hadamard operations in spatial modes ${a}_{4},{b}_{4}$

19 $$\begin{array}{ll}|{a}_{4}\rangle \to \frac{1}{\sqrt{2}}(|{a}_{4}\rangle +|{a^{\prime} }_{4}\rangle ), & |{b}_{4}\rangle \to \frac{1}{\sqrt{2}}(|{b}_{4}\rangle -|{b^{\prime} }_{4}\rangle ),\end{array}$$

where ${a^{\prime} }_{4},{b^{\prime} }_{4}$ are another two spatial modes of photon ${B}_{1}$.

After photons ${B}_{1}$ pass through $B{S}_{4},B{S}_{5}$, the state of photons ${A}_{1},{A}_{2},{B}_{2},{B}_{1}$ becomes

20 $$\begin{array}{rcl}|{\varphi }_{2}\rangle  & = & |{\varphi }_{1}^{p}\rangle ({\gamma }_{1}|{a}_{1}\rangle +{\delta }_{1}|{b}_{1}\rangle )(|{a}_{2}{a}_{3}\rangle +|{a^{\prime} }_{2}{a}_{3}\rangle +|{b}_{2}{b}_{3}\rangle \\  &  & +\,|{b^{\prime} }_{2}{b}_{3}\rangle )({\gamma ^{\prime} }_{2}|{a}_{4}\rangle +{\delta ^{\prime} }_{2}|{b}_{4}\rangle +{\gamma ^{\prime} }_{2}|{a^{\prime} }_{4}\rangle +{\delta ^{\prime} }_{2}|{b^{\prime} }_{4}\rangle ).\end{array}$$

After photons ${A}_{2}$, ${B}_{1}$ pass through $B{S}_{j},(j=1,2,3,4,5)$, Alice(Bob) let photons ${A}_{1},{A}_{2}$ $({B}_{2},{B}_{1})$ interact with probe coherent beams $|\alpha {\rangle }_{1}$ ($|\alpha {\rangle }_{2}$) via cross-Kerr nonlinearity. The state of photons ${A}_{1},{A}_{2},{B}_{2},{B}_{1}$ and two probe coherent beams $|\alpha {\rangle }_{1}$, $|\alpha {\rangle }_{2}$ can be written as:

21 $$\begin{array}{rcl}|\Phi \rangle  & = & |{\varphi }_{1}^{p}\rangle ({\gamma }_{1}|{a}_{1}\rangle +{\delta }_{1}|{b}_{1}\rangle )(|{a}_{2}{a}_{3}\rangle +|{a^{\prime} }_{2}{a}_{3}\rangle +|{b}_{2}{b}_{3}\rangle +|{b^{\prime} }_{2}{b}_{3}\rangle )\\  &  & \times \,({\gamma ^{\prime} }_{2}|{a}_{4}\rangle +{\delta ^{\prime} }_{2}|{b}_{4}\rangle +{\gamma ^{\prime} }_{2}|{a^{\prime} }_{4}\rangle +{\delta ^{\prime} }_{2}|{b^{\prime} }_{4}\rangle )|\alpha {\rangle }_{1}|\alpha {\rangle }_{2}.\end{array}$$

The state of composite system composed photons ${A}_{1},{A}_{2},{B}_{2},{B}_{1}$ and two probe coherent beams $|\alpha {\rangle }_{1}$, $|\alpha {\rangle }_{2}$ becomes

22 $$\begin{array}{rcl}|{\Phi }_{1}\rangle  & = & |{\varphi }_{1}^{p}\rangle [|{\varphi }_{3}\rangle |\alpha {\rangle }_{1}|\alpha {\rangle }_{2}+{({\sigma }_{x}^{s})}_{{a}_{4}{a^{\prime} }_{4}}{({\sigma }_{x}^{s})}_{{b}_{4}{b^{\prime} }_{4}}|{\varphi }_{3}\rangle |\alpha {\rangle }_{1}|\alpha {e}^{\pm i\theta }{\rangle }_{2}\\  &  & +\,{({\sigma }_{x}^{s})}_{{a}_{2}{a^{\prime} }_{2}}{({\sigma }_{x}^{s})}_{{b}_{2}{b^{\prime} }_{2}}|{\varphi }_{3}\rangle |\alpha {e}^{\pm i\theta }{\rangle }_{1}|\alpha {\rangle }_{2}+{({\sigma }_{x}^{s})}_{{a}_{2}{a^{\prime} }_{2}}\\  &  & \times \,{({\sigma }_{x}^{s})}_{{b}_{2}{b^{\prime} }_{2}}{({\sigma }_{x}^{s})}_{{a}_{4}{a^{\prime} }_{4}}{({\sigma }_{x}^{s})}_{{b}_{4}{b^{\prime} }_{4}}|{\varphi }_{3}\rangle |\alpha {e}^{\pm i\theta }{\rangle }_{1}|\alpha {e}^{\pm i\theta }{\rangle }_{2}]\end{array}$$

after the cross-Kerr interactions. Here

23 $$\begin{array}{rcl}|{\varphi }_{3}\rangle  & = & {\gamma }_{1}|{a}_{1}\rangle |{a}_{2}{a}_{3}\rangle ({\gamma ^{\prime} }_{2}|{a}_{4}\rangle +{\delta ^{\prime} }_{2}|{b}_{4}\rangle )+{\gamma }_{1}|{a}_{1}\rangle |{b}_{2}{b}_{3}\rangle \\  &  & \times \,({\gamma ^{\prime} }_{2}|{a^{\prime} }_{4}\rangle +{\delta ^{\prime} }_{2}|{b^{\prime} }_{4}\rangle )+{\delta }_{1}|{b}_{1}\rangle |{a^{\prime} }_{2}{a}_{3}\rangle \\  &  & \times \,({\gamma ^{\prime} }_{2}|{a}_{4}\rangle +{\delta ^{\prime} }_{2}|{b}_{4}\rangle )+{\delta }_{1}|{b}_{1}\rangle |{b^{\prime} }_{2}{b}_{3}\rangle \\  &  & \times \,({\gamma ^{\prime} }_{2}|{a^{\prime} }_{4}\rangle +{\delta ^{\prime} }_{2}|{b^{\prime} }_{4}\rangle ),\end{array}$$

Alice and Bob perform X quadrature measurements on the probe coherent beams $|\alpha {\rangle }_{1}$, $|\alpha {\rangle }_{2}$. The state of photons ${A}_{1},{A}_{2},{B}_{2},{B}_{1}$ evolves to $|{\varphi }_{3}\rangle$, ${({\sigma }_{x}^{s})}_{{a}_{4}{a^{\prime} }_{4}}{({\sigma }_{x}^{s})}_{{b}_{4}{b^{\prime} }_{4}}|{\varphi }_{3}\rangle$, ${({\sigma }_{x}^{s})}_{{a}_{2}{a^{\prime} }_{2}}{({\sigma }_{x}^{s})}_{{b}_{2}{b^{\prime} }_{2}}|{\varphi }_{3}\rangle$ or ${({\sigma }_{x}^{s})}_{{a}_{2}{a^{\prime} }_{2}}$ ${({\sigma }_{x}^{s})}_{{b}_{2}{b^{\prime} }_{2}}$ ${({\sigma }_{x}^{s})}_{{a}_{4}{a^{\prime} }_{4}}{({\sigma }_{x}^{s})}_{{b}_{4}{b^{\prime} }_{4}}|{\varphi }_{3}\rangle$ if the X quadrature measurements result is $|\alpha {\rangle }_{1}|\alpha {\rangle }_{2}$, $|\alpha {\rangle }_{1}|\alpha {e}^{\pm i\theta }{\rangle }_{2}$, $|\alpha {e}^{\pm i\theta }{\rangle }_{1}|\alpha {\rangle }_{2}$ or $|\alpha {e}^{\pm i\theta }{\rangle }_{1}|\alpha {e}^{\pm i\theta }{\rangle }_{2}$. The agents can transform the state of photons ${A}_{1},{A}_{2},{B}_{2},{B}_{1}$ to $|{\varphi }_{3}\rangle$ by performing corresponding unitary operation I, ${({\sigma }_{x}^{s})}_{{a}_{4}{a^{\prime} }_{4}}{({\sigma }_{x}^{s})}_{{b}_{4}{b^{\prime} }_{4}}$, ${({\sigma }_{x}^{s})}_{{a}_{2}{a^{\prime} }_{2}}{({\sigma }_{x}^{s})}_{{b}_{2}{b^{\prime} }_{2}}$ or ${({\sigma }_{x}^{s})}_{{a}_{2}{a^{\prime} }_{2}}{({\sigma }_{x}^{s})}_{{b}_{2}{b^{\prime} }_{2}}$ ${({\sigma }_{x}^{s})}_{{a}_{4}{a^{\prime} }_{4}}$ ${({\sigma }_{x}^{s})}_{{b}_{4}{b^{\prime} }_{4}}$ on photons ${A}_{2},{B}_{1}$ if the X quadrature measurements result of the probe coherent beams $|\alpha {\rangle }_{1}$, $|\alpha {\rangle }_{2}$ is $|\alpha {\rangle }_{1}|\alpha {\rangle }_{2}$, $|\alpha {\rangle }_{1}|\alpha {e}^{\pm i\theta }{\rangle }_{2}$, $|\alpha {e}^{\pm i\theta }{\rangle }_{1}|\alpha {\rangle }_{2}$ or $|\alpha {e}^{\pm i\theta }{\rangle }_{1}$ $|\alpha {e}^{\pm i\theta }{\rangle }_{2}$, respectively.

After transform the state of photons ${A}_{1},{A}_{2}$, ${B}_{2},{B}_{1}$ to $|{\varphi }_{3}\rangle$, Alice let photon ${A}_{2}$ interact with probe coherent beam $|\alpha {\rangle }_{3}$ via cross-Kerr nonlinearity. The state of photons ${A}_{1},{A}_{2}$, ${B}_{2},{B}_{1}$ and probe coherent beam $|{\varphi }_{3}\rangle$ can be written as

24 $$\begin{array}{rcl}|{\Phi }_{2}\rangle  & = & [{\gamma }_{1}|{a}_{1}\rangle |{a}_{2}{a}_{3}\rangle ({\gamma ^{\prime} }_{2}|{a}_{4}\rangle +{\delta ^{\prime} }_{2}|{b}_{4}\rangle )+{\gamma }_{1}|{a}_{1}\rangle |{b}_{2}{b}_{3}\rangle \\  &  & \times \,({\gamma ^{\prime} }_{2}|{a^{\prime} }_{4}\rangle +{\delta ^{\prime} }_{2}|{b^{\prime} }_{4}\rangle )+{\delta }_{1}|{b}_{1}\rangle |{a^{\prime} }_{2}{a}_{3}\rangle \\  &  & \times \,({\gamma ^{\prime} }_{2}|{a}_{4}\rangle +{\delta ^{\prime} }_{2}|{b}_{4}\rangle )+{\delta }_{1}|{b}_{1}\rangle |{b^{\prime} }_{2}{b}_{3}\rangle \\  &  & \times \,({\gamma ^{\prime} }_{2}|{a^{\prime} }_{4}\rangle +{\delta ^{\prime} }_{2}|{b^{\prime} }_{4}\rangle )]|\alpha {\rangle }_{3}.\end{array}$$

The state of composite system composed of photons ${A}_{1},{A}_{2}$, ${B}_{2},{B}_{1}$ and probe coherent beam evolves to

25 $$\begin{array}{rcl}|{\Phi }_{3}\rangle  & = & [{\gamma }_{1}|{a}_{1}\rangle |{a}_{2}{a}_{3}\rangle ({\gamma ^{\prime} }_{2}|{a}_{4}\rangle +{\delta ^{\prime} }_{2}|{b}_{4}\rangle )+{\delta }_{1}|{b}_{1}\rangle |{b^{\prime} }_{2}{b}_{3}\rangle \\  &  & \times \,({\gamma ^{\prime} }_{2}|{a^{\prime} }_{4}\rangle +{\delta ^{\prime} }_{2}|{b^{\prime} }_{4}\rangle )]|\alpha {e}^{i\theta }{\rangle }_{3}\\  &  & +\,[{\gamma }_{1}|{a}_{1}\rangle |{b}_{2}{b}_{3}\rangle ({\gamma ^{\prime} }_{2}|{a^{\prime} }_{4}\rangle +{\delta ^{\prime} }_{2}|{b^{\prime} }_{4}\rangle )\\  &  & +\,{\delta }_{1}|{b}_{1}\rangle |{a^{\prime} }_{2}{a}_{3}\rangle ({\gamma ^{\prime} }_{2}|{a}_{4}\rangle +{\delta ^{\prime} }_{2}|{b}_{4}\rangle )|\alpha {\rangle }_{3}]\end{array}$$

after the corss-Kerr interaction between photon ${A}_{2}$ and the probe coherent beam $|\alpha {\rangle }_{3}$.

The state of photons ${A}_{1},{A}_{2}$, ${B}_{2},{B}_{1}$ evolves to $|{\varphi }_{4}\rangle$ or $|{\varphi ^{\prime} }_{4}\rangle$ if the X quadrature measurement result of the probe coherent beam is $|\alpha {e}^{i\theta }{\rangle }_{3}$ or $|\alpha {\rangle }_{3}$. Here

26 $$\begin{array}{rcl}|{\varphi }_{4}\rangle  & = & {\gamma }_{1}|{a}_{1}\rangle |{a}_{2}{a}_{3}\rangle ({\gamma ^{\prime} }_{2}|{a}_{4}\rangle +{\delta ^{\prime} }_{2}|{b}_{4}\rangle )\\  &  & +\,{\delta }_{1}|{b}_{1}\rangle |{b^{\prime} }_{2}{b}_{3}\rangle ({\gamma ^{\prime} }_{2}|{a^{\prime} }_{4}\rangle +{\delta ^{\prime} }_{2}|{b^{\prime} }_{4}\rangle ),\\ |{\varphi ^{\prime} }_{4}\rangle  & = & {\gamma }_{1}|{a}_{1}\rangle |{b}_{2}{b}_{3}\rangle ({\gamma ^{\prime} }_{2}|{a^{\prime} }_{4}\rangle +{\delta ^{\prime} }_{2}|{b^{\prime} }_{4}\rangle )\\  &  & +\,{\delta }_{1}|{b}_{1}\rangle |{a^{\prime} }_{2}{a}_{3}\rangle ({\gamma ^{\prime} }_{2}|{a}_{4}\rangle +{\delta ^{\prime} }_{2}|{b}_{4}\rangle ).\end{array}$$

Suppose the X quadrature measurement result is $|\alpha {e}^{i\theta }{\rangle }_{3}$, the state of photons becomes $|{\varphi }_{4}\rangle$. To implement the CNOT operation nonlocally, Bob applies -I operation in spatial mode ${b^{\prime} }_{4}$, let photon ${B}_{1}$ pass through $B{S}_{k}$ $(k=6,7,8,9)$ and interact with the probe coherent beam $|\alpha {\rangle }_{4}$. After Bob applies -I operation in spatial mode ${b^{\prime} }_{4}$, the state of four photons evolves to

27 $$\begin{array}{rcl}|{\varphi }_{5}\rangle  & = & {\gamma }_{1}|{a}_{1}\rangle |{a}_{2}{a}_{3}\rangle ({\gamma ^{\prime} }_{2}|{a}_{4}\rangle +{\delta ^{\prime} }_{2}|{b}_{4}\rangle )\\  &  & +\,{\delta }_{1}|{b}_{1}\rangle |{b^{\prime} }_{2}{b}_{3}\rangle ({\gamma ^{\prime} }_{2}|{a^{\prime} }_{4}\rangle -{\delta ^{\prime} }_{2}|{b^{\prime} }_{4}\rangle ).\end{array}$$

After the -I operation, Bob let photon ${B}_{1}$ pass through $B{S}_{6},B{S}_{7}$ which implement Hadamard operation in spatial modes ${a}_{4},{b}_{4},{a^{\prime} }_{4},{b^{\prime} }_{4}$

28 $$\begin{array}{ll}|{a}_{4}\rangle \to \frac{1}{\sqrt{2}}(|{a}_{4}\rangle +|{b}_{4}\rangle ), & |{b}_{4}\rangle \to \frac{1}{\sqrt{2}}(|{a}_{4}\rangle -|{b}_{4}\rangle ),\\ |{a^{\prime} }_{4}\rangle \to \frac{1}{\sqrt{2}}(|{a^{\prime} }_{4}\rangle +|{b^{\prime} }_{4}\rangle ), & |{b^{\prime} }_{4}\rangle \to \frac{1}{\sqrt{2}}(|{a^{\prime} }_{4}\rangle -|{b^{\prime} }_{4}\rangle ).\end{array}$$

The state of four photons becomes

29 $$\begin{array}{rcl}|{\varphi }_{6}\rangle  & = & {\gamma }_{1}|{a}_{1}\rangle |{a}_{2}{a}_{3}\rangle ({\gamma }_{2}|{a}_{4}\rangle +{\delta }_{2}|{b}_{4}\rangle )\\  &  & +\,{\delta }_{1}|{b}_{1}\rangle |{b^{\prime} }_{2}{b}_{3}\rangle ({\delta }_{2}|{a^{\prime} }_{4}\rangle +{\gamma }_{2}|{b^{\prime} }_{4}\rangle ).\end{array}$$

For implementation of the nonlocal CNOT operation in spatial-mode DOF, Bob let photon ${B}_{1}$ pass through $B{S}_{6},B{S}_{7}$ which implement Hadamard operation in spatial mode

30 $$\begin{array}{ll}|{a}_{4}\rangle \to \frac{1}{\sqrt{2}}(|{a}_{4}\rangle +|{a^{\prime} }_{4}\rangle ), & |{a^{\prime} }_{4}\rangle \to \frac{1}{\sqrt{2}}(|{a}_{4}\rangle -|{a^{\prime} }_{4}\rangle ),\\ |{b}_{4}\rangle \to \frac{1}{\sqrt{2}}(|{b}_{4}\rangle +|{b^{\prime} }_{4}\rangle ), & |{b^{\prime} }_{4}\rangle \to \frac{1}{\sqrt{2}}(|{b}_{4}\rangle -|{b^{\prime} }_{4}\rangle ).\end{array}$$

After photon ${B}_{1}$ pass through $B{S}_{6},B{S}_{7}$, the state of photons becomes

31 $$\begin{array}{rcl}|{\varphi }_{7}\rangle  & = & {\gamma }_{1}|{a}_{1}\rangle |{a}_{2}{a}_{3}\rangle ({\gamma }_{2}|{a}_{4}\rangle +{\delta }_{2}|{b}_{4}\rangle +{\gamma }_{2}|{a^{\prime} }_{4}\rangle +{\delta }_{2}|{b^{\prime} }_{4}\rangle )\\  &  & +\,{\delta }_{1}|{b}_{1}\rangle |{b^{\prime} }_{2}{b}_{3}\rangle ({\delta }_{2}|{a}_{4}\rangle +{\gamma }_{2}|{b}_{4}-\rangle {\delta }_{2}|{a^{\prime} }_{4}\rangle -{\gamma }_{2}|{b^{\prime} }_{4}\rangle ).\end{array}$$

Bob let photon ${B}_{1}$ interact with the probe coherent beam $|\alpha {\rangle }_{4}$ via cross-Kerr nonlinearity. The state of photons ${A}_{1},{A}_{2}$, ${B}_{2},{B}_{1}$ and probe coherent beam can be written as^67,68^

32 $$\begin{array}{rcl}|{\Phi }_{4}\rangle  & = & {\gamma }_{1}|{a}_{1}\rangle |{a}_{2}{a}_{3}\rangle ({\gamma }_{2}|{a}_{4}\rangle +{\delta }_{2}|{b}_{4}\rangle +{\gamma }_{2}|{a^{\prime} }_{4}\rangle +{\delta }_{2}|{b^{\prime} }_{4}\rangle )\\  &  & +\,{\delta }_{1}|{b}_{1}\rangle |{b^{\prime} }_{2}{b}_{3}\rangle ({\delta }_{2}|{a}_{4}\rangle +{\gamma }_{2}|{b}_{4}-\rangle {\delta }_{2}|{a^{\prime} }_{4}\rangle -{\gamma }_{2}|{b^{\prime} }_{4}\rangle )|\alpha {\rangle }_{4}.\end{array}$$

After the corss-Kerr nonlinearity, the state of composite system becomes

33 $$|{\Phi }_{5}\rangle =|{\varphi }_{8}\rangle |\alpha {\rangle }_{4}+{({\sigma }_{z}^{s})}_{{A}_{1}}|{\varphi }_{8}\rangle |\alpha {e}^{i\theta }{\rangle }_{4},$$

where ${({\sigma }_{z}^{s})}_{{A}_{1}}=|{a}_{1}\rangle \langle {a}_{1}|-|{b}_{1}\rangle \langle {b}_{1}|$ represents phase-flip operation in spatial-mode DOF, and

34 $$|{\varphi }_{8}\rangle ={\gamma }_{1}|{a}_{1}\rangle |{a}_{2}{a}_{3}\rangle ({\gamma }_{2}|{a}_{4}\rangle +{\delta }_{2}|{b}_{4}\rangle )+{\delta }_{1}|{b}_{1}\rangle |{b^{\prime} }_{2}{b}_{3}\rangle ({\delta }_{2}|{a}_{4}\rangle +{\gamma }_{2}|{b}_{4}).$$

The state of four photons becomes $|{\varphi }_{8}\rangle$ or ${({\sigma }_{z}^{s})}_{{A}_{1}}|{\varphi }_{8}\rangle$ if the X quadrature measurement result is $|\alpha {\rangle }_{4}$ or $|\alpha {e}^{i\theta }{\rangle }_{4}$. Alice can transform the spatial-mode state of photons ${A}_{1},{A}_{2},{B}_{2},{B}_{1}$ to $|{\varphi }_{8}\rangle$ by performing unitary operation I or ${({\sigma }_{z}^{s})}_{{A}_{1}}$ on photon ${A}_{1}$ if the measurement result is $|\alpha {\rangle }_{4}$ or $|\alpha {e}^{i\theta }{\rangle }_{4}$. After transform the spatial-mode state of photons ${A}_{1},{A}_{2},{B}_{2},{B}_{1}$ to $|{\varphi }_{8}\rangle$, the state of photons ${A}_{1},{A}_{2},{B}_{2},{B}_{1}$ can be written as

35 $$\begin{array}{rcl}|\phi \rangle  & = & |{\varphi }_{1}^{p}\rangle \otimes |{\varphi }_{8}\rangle \\  & = & [{\alpha }_{1}|H\rangle |HH\rangle ({\alpha }_{2}|H\rangle +{\beta }_{2}|V\rangle )+{\alpha }_{1}|H\rangle |VV\rangle \\  &  & \times \,({\beta }_{2}|H\rangle +{\alpha }_{2}|V\rangle )+{\beta }_{1}|V\rangle |VH\rangle \\  &  & \times \,({\alpha }_{2}|H\rangle +{\beta }_{2}|V\rangle )+{\beta }_{1}|V\rangle |HV\rangle \\  &  & \times \,({\beta }_{2}|H\rangle +{\alpha }_{2}|V\rangle )]\otimes [{\gamma }_{1}|{a}_{1}\rangle |{a}_{2}{a}_{3}\rangle \\  &  & \times \,({\gamma }_{2}|{a}_{4}\rangle +{\delta }_{2}|{b}_{4}\rangle )+{\delta }_{1}|{b}_{1}\rangle |{b^{\prime} }_{2}{b}_{3}\rangle ({\delta }_{2}|{a}_{4}\rangle +{\gamma }_{2}|{b}_{4})].\end{array}$$

To implement CNOT in polarization and spatial-mode DOFs, Alice and Bob perform single-qubit measurements on photons ${A}_{2}$, ${B}_{2}$ and apply corresponding operations on photons ${A}_{1}$, ${B}_{1}$. That is, Alice lets photons ${A}_{2}$ pass through $B{S}_{10}$ which implement Hadamard operation in saptial modes ${a}_{2},{b^{\prime} }_{2}$. Bob first lets photon ${B}_{2}$ pass through $B{S}_{11}$ which implement Hadamard operation in saptial modes ${a}_{3},{b}_{3}$, then lets photons ${B}_{2}$ pass through wave plates ${R}_{45}$ in spatial modes ${a}_{3},{b}_{3}$ which implement Hadamard operations in polarization DOF

36 $$\begin{array}{ll}|{a}_{2}\rangle \to \frac{1}{\sqrt{2}}(|{a}_{2}\rangle +|{b^{\prime} }_{2}\rangle ), & |{b^{\prime} }_{2}\rangle \to \frac{1}{\sqrt{2}}(|{a}_{2}\rangle -|{b^{\prime} }_{2}\rangle ),\\ |{a}_{3}\rangle \to \frac{1}{\sqrt{2}}(|{a}_{3}\rangle +|{b}_{3}\rangle ), & |{b}_{3}\rangle \to \frac{1}{\sqrt{2}}(|{a}_{3}\rangle -|{b}_{3}\rangle ).\end{array}$$

After photons ${A}_{2},{B}_{2}$ pass through $B{S}_{10}$, $B{S}_{11}$ and wave plates ${R}_{45}$, the state of photons ${A}_{1},{A}_{2},{B}_{2},{B}_{1}$ evolves to $|{\phi }_{1}\rangle$

37 $$\begin{array}{rcl}|{\phi }_{1}\rangle  & = & [{\alpha }_{1}|H\rangle |H\rangle (|H\rangle +|V\rangle )({\alpha }_{2}|H\rangle +{\beta }_{2}|V\rangle )+{\alpha }_{1}|H\rangle |V\rangle \\  &  & \times \,(|H\rangle -|V\rangle )({\beta }_{2}|H\rangle +{\alpha }_{2}|V\rangle )+{\beta }_{1}|V\rangle |V\rangle (|H\rangle +|V\rangle )\\  &  & \times \,({\alpha }_{2}|H\rangle +{\beta }_{2}|V\rangle )+{\beta }_{1}|V\rangle |H\rangle (|H\rangle -|V\rangle )\\  &  & \times \,({\beta }_{2}|H\rangle +{\alpha }_{2}|V\rangle )]\otimes [{\gamma }_{1}|{a}_{1}\rangle (|{a}_{2}\rangle +|{b^{\prime} }_{2}\rangle )\\  &  & \times \,(|{a}_{3}\rangle +|{b}_{3}\rangle )({\gamma }_{2}|{a}_{4}\rangle +{\delta }_{2}|{b}_{4}\rangle )+{\delta }_{1}|{b}_{1}\rangle \\  &  & \times \,(|{a}_{2}\rangle -|{b^{\prime} }_{2}\rangle )(|{a}_{3}\rangle -|{b}_{3}\rangle )({\delta }_{2}|{a}_{4}\rangle +{\gamma }_{2}|{b}_{4})].\end{array}$$

The state of photons ${A}_{1},{A}_{2},{B}_{2},{B}_{1}$ can be rewritten as:

38 $$\begin{array}{rcl}|{\phi }_{1}\rangle  & = & |H{a}_{2}\rangle |H{a}_{3}\rangle |\xi \rangle +|H{a}_{2}\rangle |H{b}_{3}\rangle {({\sigma }_{z}^{s})}_{{A}_{1}}|\xi \rangle |H{a}_{2}\rangle |V{a}_{3}\rangle \\  &  & \times \,{({\sigma }_{z}^{p})}_{{A}_{1}}|\xi \rangle +|H{a}_{2}\rangle |V{b}_{3}\rangle {({\sigma }_{z}^{p})}_{{A}_{1}}{({\sigma }_{z}^{s})}_{{A}_{1}}|\xi \rangle +|H{b^{\prime} }_{2}\rangle |H{a}_{3}\rangle \\  &  & \times \,{({\sigma }_{z}^{s})}_{{A}_{1}}|\xi \rangle +|H{b^{\prime} }_{2}\rangle |H{b}_{3}\rangle |\xi \rangle +|H{b^{\prime} }_{2}\rangle |V{a}_{3}\rangle {({\sigma }_{z}^{p})}_{{A}_{1}}{({\sigma }_{z}^{s})}_{{A}_{1}}|\xi \rangle \\  &  & +\,|H{b^{\prime} }_{2}\rangle |V{b}_{3}\rangle {({\sigma }_{z}^{p})}_{{A}_{1}}|\xi \rangle +|V{a}_{2}\rangle |H{a}_{3}\rangle {({\sigma }_{x}^{p})}_{{A}_{2}}|\xi \rangle \\  &  & +\,|V{a}_{2}\rangle |H{b}_{3}\rangle {({\sigma }_{x}^{p})}_{{B}_{1}}{({\sigma }_{z}^{s})}_{{A}_{1}}|\xi \rangle +|V{a}_{2}\rangle |V{a}_{3}\rangle {({\sigma }_{x}^{p})}_{{B}_{1}}\\  &  & \times \,{({\sigma }_{z}^{p})}_{{A}_{1}}|\xi \rangle +|V{a}_{2}\rangle |V{b}_{3}\rangle {({\sigma }_{x}^{p})}_{{B}_{1}}{({\sigma }_{z}^{p})}_{{A}_{1}}{({\sigma }_{z}^{s})}_{{A}_{1}}|\xi \rangle \\  &  & +\,|V{b^{\prime} }_{2}\rangle |H{a}_{3}\rangle {({\sigma }_{x}^{p})}_{{B}_{1}}{({\sigma }_{z}^{s})}_{{A}_{1}}|\xi \rangle +|V{b^{\prime} }_{2}\rangle |H{b}_{3}\rangle {({\sigma }_{x}^{p})}_{{B}_{1}}|\xi \rangle \\  &  & +\,|V{b^{\prime} }_{2}\rangle |V{a}_{3}\rangle {({\sigma }_{x}^{p})}_{{B}_{1}}{({\sigma }_{z}^{s})}_{{A}_{1}}{({\sigma }_{z}^{p})}_{{A}_{1}}|\xi \rangle \\  &  & +\,|V{b^{\prime} }_{2}\rangle |V{b}_{3}\rangle {({\sigma }_{x}^{p})}_{{B}_{1}}{({\sigma }_{z}^{p})}_{{A}_{1}}|\xi \rangle .\end{array}$$

Here

39 $$\begin{array}{rcl}|\xi \rangle  & = & [{\alpha }_{1}|H\rangle ({\alpha }_{2}|H\rangle +{\beta }_{2}|V\rangle )+{\beta }_{1}|V\rangle \\  &  & \times \,({\beta }_{2}|H\rangle +{\alpha }_{2}|V\rangle )]\otimes [{\gamma }_{1}|{a}_{1}\rangle ({\gamma }_{2}|{a}_{4}\rangle +{\delta }_{2}|{b}_{4}\rangle )\\  &  & +\,{\delta }_{1}|{b}_{1}\rangle ({\delta }_{2}|{a}_{4}\rangle +{\gamma }_{2}|{b}_{4})].\end{array}$$

The nonlocal CNOT operation in spatial-mode DOF can be realized remotely by applying -I operation in spatial mode ${b^{\prime} }_{4}$, implementing Hadamard operation in spatial-mode DOF via BSs, introducing probe coherent beams and performing corresponding unitary operations on photons ${A}_{1}$ when the X quadrature measurement result is $|\alpha {e}^{i\theta }{\rangle }_{3}$. Similar to the case of $|\alpha {e}^{i\theta }{\rangle }_{3}$, the nonlocal CNOT operation can be remote realized by applying -I operation in spatial mode ${b}_{4}$, implementing Hadamard operation in spatial-mode DOF, interacting photon ${B}_{1}$ with probe coherent beam via cross-Kerr nonlinearity and performing corresponding unitary according to the measurement result when the X quadrature measurement result is $|\alpha {\rangle }_{3}$. The relation between the measurement results of photons ${A}_{2},{B}_{2}$, the state of photons ${A}_{1},{B}_{1}$ after the measurements and the recovery operation with which Alice and Bob can transform the state of photons ${A}_{1},{B}_{1}$ to the target state $|\xi \rangle$ is shown in Table 3.

**Table 3:** The relation between the measurement results of photons ${A}_{2},{B}_{2}$, the state of photons ${A}_{1},{B}_{1}$ after the measurements and the recovery operation with which Alice and Bob can transform the state of photons ${A}_{1},{B}_{1}$ to the target state.

*R*_3_	${{\boldsymbol{R}}}_{{{\boldsymbol{A}}}_{{\bf{2}}}}$	${{\boldsymbol{R}}}_{{{\boldsymbol{B}}}_{{\bf{2}}}}$	State of photos *A*_1_, *B*_1_	Recovery operation according to the measurement results
$|\alpha {e}^{i\theta }{\rangle }_{3}$	$|H{a}_{2}\rangle$	$|H{a}_{3}\rangle$	$|\xi \rangle$	*I*
$|\alpha {e}^{i\theta }{\rangle }_{3}$	$|H{a}_{2}\rangle$	$|H{b}_{3}\rangle$	${({\sigma }_{z}^{s})}_{{A}_{1}}|\xi \rangle$	${({\sigma }_{z}^{s})}_{{A}_{1}}$
$|\alpha {e}^{i\theta }{\rangle }_{3}$	$|H{a}_{2}\rangle$	$|V{a}_{3}\rangle$	${({\sigma }_{z}^{p})}_{{A}_{1}}|\xi \rangle$	${({\sigma }_{z}^{p})}_{{A}_{1}}$
$|\alpha {e}^{i\theta }{\rangle }_{3}$	$|H{a}_{2}\rangle$	$|V{b}_{3}\rangle$	${({\sigma }_{z}^{p})}_{{A}_{1}}{({\sigma }_{z}^{s})}_{{A}_{1}}|\xi \rangle$	${({\sigma }_{z}^{p})}_{{A}_{1}}{({\sigma }_{z}^{s})}_{{A}_{1}}$
$|\alpha {e}^{i\theta }{\rangle }_{3}$	$|H{b^{\prime} }_{2}\rangle$	$|H{a}_{3}\rangle$	${({\sigma }_{z}^{s})}_{{A}_{1}}|\xi \rangle$	${({\sigma }_{z}^{s})}_{{A}_{1}}$
$|\alpha {e}^{i\theta }{\rangle }_{3}$	$|H{b^{\prime} }_{2}\rangle$	$|H{b}_{3}\rangle$	$|\xi \rangle$	*I*
$|\alpha {e}^{i\theta }{\rangle }_{3}$	$|H{b^{\prime} }_{2}\rangle$	$|V{a}_{3}\rangle$	${({\sigma }_{z}^{p})}_{{A}_{1}}{({\sigma }_{z}^{s})}_{{A}_{1}}|\xi \rangle$	${({\sigma }_{z}^{p})}_{{A}_{1}}{({\sigma }_{z}^{s})}_{{A}_{1}}$
$|\alpha {e}^{i\theta }{\rangle }_{3}$	$|H{b^{\prime} }_{2}\rangle$	$|V{b}_{3}\rangle$	${({\sigma }_{z}^{p})}_{{A}_{1}}|\xi \rangle$	${({\sigma }_{z}^{p})}_{{A}_{1}}$
$|\alpha {e}^{i\theta }{\rangle }_{3}$	$|V{a}_{2}\rangle$	$|H{a}_{3}\rangle$	${({\sigma }_{x}^{p})}_{{B}_{1}}|\xi \rangle$	${({\sigma }_{x}^{p})}_{{B}_{1}}$
$|\alpha {e}^{i\theta }{\rangle }_{3}$	$|V{a}_{2}\rangle$	$|H{b}_{3}\rangle$	${({\sigma }_{x}^{p})}_{{B}_{1}}{({\sigma }_{z}^{s})}_{{A}_{1}}|\xi \rangle$	${({\sigma }_{x}^{p})}_{{B}_{1}}{({\sigma }_{z}^{s})}_{{A}_{1}}$
$|\alpha {e}^{i\theta }{\rangle }_{3}$	$|V{a}_{2}\rangle$	$|V{a}_{3}\rangle$	${({\sigma }_{x}^{p})}_{{B}_{1}}{({\sigma }_{z}^{p})}_{{A}_{1}}|\xi \rangle$	${({\sigma }_{x}^{p})}_{{B}_{1}}{({\sigma }_{z}^{p})}_{{A}_{1}}$
$|\alpha {e}^{i\theta }{\rangle }_{3}$	$|V{a}_{2}\rangle$	$|V{b}_{3}\rangle$	${({\sigma }_{x}^{p})}_{{B}_{1}}{({\sigma }_{z}^{p})}_{{A}_{1}}{({\sigma }_{z}^{s})}_{{A}_{1}}|\xi \rangle$	${({\sigma }_{x}^{p})}_{{B}_{1}}{({\sigma }_{z}^{p})}_{{A}_{1}}{({\sigma }_{z}^{s})}_{{A}_{1}}$
$|\alpha {e}^{i\theta }{\rangle }_{3}$	$|V{b^{\prime} }_{2}\rangle$	$|H{a}_{3}\rangle$	${({\sigma }_{x}^{p})}_{{B}_{1}}{({\sigma }_{z}^{s})}_{{A}_{1}}|\xi \rangle$	${({\sigma }_{x}^{p})}_{{B}_{1}}{({\sigma }_{z}^{s})}_{{A}_{1}}$
$|\alpha {e}^{i\theta }{\rangle }_{3}$	$|V{b^{\prime} }_{2}\rangle$	$|H{b}_{3}\rangle$	${({\sigma }_{x}^{p})}_{{B}_{1}}|\xi \rangle$	${({\sigma }_{x}^{p})}_{{B}_{1}}$
$|\alpha {e}^{i\theta }{\rangle }_{3}$	$|V{b^{\prime} }_{2}\rangle$	$|V{a}_{3}\rangle$	${({\sigma }_{x}^{p})}_{{B}_{1}}{({\sigma }_{z}^{p})}_{{A}_{1}}{({\sigma }_{z}^{s})}_{{A}_{1}}|\xi \rangle$	${({\sigma }_{x}^{p})}_{{B}_{1}}{({\sigma }_{z}^{p})}_{{A}_{1}}{({\sigma }_{z}^{s})}_{{A}_{1}}$
$|\alpha {e}^{i\theta }{\rangle }_{3}$	$|V{b^{\prime} }_{2}\rangle$	$|V{b}_{3}\rangle$	${({\sigma }_{x}^{p})}_{{B}_{1}}{({\sigma }_{z}^{p})}_{{A}_{1}}|\xi \rangle$	${({\sigma }_{x}^{p})}_{{B}_{1}}{({\sigma }_{z}^{p})}_{{A}_{1}}$
$|\alpha {\rangle }_{3}$	$|H{a^{\prime} }_{2}\rangle$	$|H{a}_{3}\rangle$	$|\xi \rangle$	*I*
$|\alpha {\rangle }_{3}$	$|H{a^{\prime} }_{2}\rangle$	$|H{b}_{3}\rangle$	${({\sigma }_{z}^{s})}_{{A}_{1}}|\xi \rangle$	${({\sigma }_{z}^{s})}_{{A}_{1}}$
$|\alpha {\rangle }_{3}$	$|H{a^{\prime} }_{2}\rangle$	$|V{a}_{3}\rangle$	${({\sigma }_{z}^{p})}_{{A}_{1}}|\xi \rangle$	${({\sigma }_{z}^{p})}_{{A}_{1}}$
$|\alpha {\rangle }_{3}$	$|H{a^{\prime} }_{2}\rangle$	$|V{b}_{3}\rangle$	${({\sigma }_{z}^{p})}_{{A}_{1}}{({\sigma }_{z}^{s})}_{{A}_{1}}|\xi \rangle$	${({\sigma }_{z}^{p})}_{{A}_{1}}{({\sigma }_{z}^{s})}_{{A}_{1}}$
$|\alpha {\rangle }_{3}$	$|H{b}_{2}\rangle$	$|H{a}_{3}\rangle$	${({\sigma }_{z}^{s})}_{{A}_{1}}|\xi \rangle$	${({\sigma }_{z}^{s})}_{{A}_{1}}$
$|\alpha {\rangle }_{3}$	$|H{b}_{2}\rangle$	$|H{b}_{3}\rangle$	$|\xi \rangle$	*I*
$|\alpha {\rangle }_{3}$	$|H{b}_{2}\rangle$	$|V{a}_{3}\rangle$	${({\sigma }_{z}^{p})}_{{A}_{1}}{({\sigma }_{z}^{s})}_{{A}_{1}}|\xi \rangle$	${({\sigma }_{z}^{p})}_{{A}_{1}}{({\sigma }_{z}^{s})}_{{A}_{1}}$
$|\alpha {\rangle }_{3}$	$|H{b}_{2}\rangle$	$|V{b}_{3}\rangle$	${({\sigma }_{z}^{p})}_{{A}_{1}}|\xi \rangle$	${({\sigma }_{z}^{p})}_{{A}_{1}}$
$|\alpha {\rangle }_{3}$	$|V{a^{\prime} }_{2}\rangle$	$|H{a}_{3}\rangle$	${({\sigma }_{x}^{p})}_{{B}_{1}}|\xi \rangle$	${({\sigma }_{x}^{p})}_{{B}_{1}}$
$|\alpha {\rangle }_{3}$	$|V{a^{\prime} }_{2}\rangle$	$|H{b}_{3}\rangle$	${({\sigma }_{x}^{p})}_{{B}_{1}}{({\sigma }_{z}^{s})}_{{A}_{1}}|\xi \rangle$	${({\sigma }_{x}^{p})}_{{B}_{1}}{({\sigma }_{z}^{s})}_{{A}_{1}}$
$|\alpha {\rangle }_{3}$	$|V{a^{\prime} }_{2}\rangle$	$|V{a}_{3}\rangle$	${({\sigma }_{x}^{p})}_{{B}_{1}}{({\sigma }_{z}^{p})}_{{A}_{1}}|\xi \rangle$	${({\sigma }_{x}^{p})}_{{B}_{1}}{({\sigma }_{z}^{p})}_{{A}_{1}}$
$|\alpha {\rangle }_{3}$	$|V{a^{\prime} }_{2}\rangle$	$|V{b}_{3}\rangle$	${({\sigma }_{x}^{p})}_{{B}_{1}}{({\sigma }_{z}^{p})}_{{A}_{1}}{({\sigma }_{z}^{s})}_{{A}_{1}}|\xi \rangle$	${({\sigma }_{x}^{p})}_{{B}_{1}}{({\sigma }_{z}^{p})}_{{A}_{1}}{({\sigma }_{z}^{s})}_{{A}_{1}}$
$|\alpha {\rangle }_{3}$	$|V{b}_{2}\rangle$	$|H{a}_{3}\rangle$	${({\sigma }_{x}^{p})}_{{B}_{1}}{({\sigma }_{z}^{s})}_{{A}_{1}}|\xi \rangle$	${({\sigma }_{x}^{p})}_{{B}_{1}}{({\sigma }_{z}^{s})}_{{A}_{1}}$
$|\alpha {\rangle }_{3}$	$|V{b}_{2}\rangle$	$|H{b}_{3}\rangle$	${({\sigma }_{x}^{p})}_{{B}_{1}}|\xi \rangle$	${({\sigma }_{x}^{p})}_{{B}_{1}}$
$|\alpha {\rangle }_{3}$	$|V{b}_{2}\rangle$	$|V{a}_{3}\rangle$	${({\sigma }_{x}^{p})}_{{B}_{1}}{({\sigma }_{z}^{p})}_{{A}_{1}}{({\sigma }_{z}^{s})}_{{A}_{1}}|\xi \rangle$	${({\sigma }_{x}^{p})}_{{B}_{1}}{({\sigma }_{z}^{p})}_{{A}_{1}}{({\sigma }_{z}^{s})}_{{A}_{1}}$
$|\alpha {\rangle }_{3}$	$|V{b}_{2}\rangle$	$|V{b}_{3}\rangle$	${({\sigma }_{x}^{p})}_{{B}_{1}}{({\sigma }_{z}^{p})}_{{A}_{1}}|\xi \rangle$	${({\sigma }_{x}^{p})}_{{B}_{1}}{({\sigma }_{z}^{p})}_{{A}_{1}}$

${R}_{{A}_{2}}$, ${R}_{{B}_{2}}$ represent the single-qubit measurement results of photons *A*_2_, *B*_2_.

## Discussion

Up to now, the utilization of cross-Kerr nonlinearities has been widely considered in the implementation of quantum information processing both theoretically and experimentally^73–79^. In 2003, Hofmann *et al*. showed than phase shift *π* can be obtained via single two-level atom in one-side cavity^73^. In 2013, Hoi *et al*. demonstrated cross-Kerr nonlinearity up to 20 degrees per photon at the single-photon level via superconducting qubit^74^. In 2016, Brod and Combes showed that cross-Kerr nonlinearity can be used to construct controlled-phase gate^75^. In addition, our protocol for hyper-parllel nonlocal CNOT operation requires only small phase shift as long as it can be distinguished from zero which makes our protocol for nonlocal CNOT operation in two DOFs more convenient in application than others.

In summary, we have proposed a protocol for parallel nonlocal implementation of CNOT operation in polarization and spatial-mode DOFs simultaneously. Assisted by cross-Kerr nonlinearity, hyperentangled state, the CNOT operation is teleported from implementing on local qubits in 2 DOFs to implementing on remote qubits. The agents first implement nonlocal CNOT operation in polarization via polarization entanglement of the hyperentangled state, then apply nonlocal CNOT operation in spatial-mode DOF via spatial-mode entanglement of the hyperentangled state. It is shown that the protocol for hyper-parallel nonlocal CNOT operation can enhance the communication for distributed quantum computation and large-scale quantum network. If large number of qubits are stored and manipulated in distributed quantum systems connected by entangled channel, nonlocal CNOT operation is required to implement distributed quantum computation. In hyper-parallel nonlocal CNOT operation, nonlocal CNOT operations are nonlocal implemented simultaneously in two DOFs which can enhance the channel capacity for large-scale quantum communication. Therefore, our protocol may be useful for large-scale quantum computation assisted by hyperentangled states.

## Methods

### The cross-Kerr nonlinearity

The cross-Kerr nonlinearity between the signal state $|n\rangle$ and the coherent state $|\alpha \rangle$ can be described by the Hamiltonian ${H}_{ck}=\hslash \chi {a}_{s}^{\dagger }{a}_{s}{a}_{c}^{\dagger }{a}_{c}$^67,68,80–82^. Here ${a}_{s}^{\dagger }$ and ${a}_{c}^{\dagger }$ represent the creation operators for signal and probe coherent states. ${a}_{s}$ and ${a}_{c}$ are the annihilation operators for the signal and probe coherent states. The coupling strength of the cross-Kerr nonlinearity is determined by the materia. After the cross-Kerr nonlinearity interaction between the signal state $|n\rangle$ and the coherent state $|\alpha \rangle$, the probe coherent state has a phase shift which is proportional with the number of photons n in the signal state $|n\rangle$.

40 $${H}_{ck}|n\rangle |\alpha \rangle =|n\rangle |\alpha {e}^{in\theta }\rangle .$$

where $\theta =\chi t$ and t represents the interaction time of cross-Kerr nonlinearity. The signal state is unchanged after the cross-Kerr nonlinearity and the coherent probe state picks up a phase shift ${e}^{in\theta }$. The number of photons in signal state can be determined without destroying the signal state via cross-Kerr nonlinearity since the phase shift ${e}^{in\theta }$ can be detected by the homodyne measurement.
